# Fetuin-A alleviates neuroinflammation against traumatic brain injury-induced microglial necroptosis by regulating Nrf-2/HO-1 pathway

**DOI:** 10.1186/s12974-022-02633-5

**Published:** 2022-11-04

**Authors:** Pengzhan Zhao, Yutian Wei, Guangchi Sun, Lei Xu, Tian Wang, Yufei Tian, Honglu Chao, Yiming Tu, Jing Ji

**Affiliations:** 1 Department of Neurosurgery, The First Affiliated Hospital of Nanjing Medical University, Nanjing, 210029 Jiangsu China; 2 Department of Neurosurgery, The First Affiliated Hospital of Anhui University of Chinese Medicine, Hefei, 230031 Anhui China; 3 Gusu School, Nanjing Medical University, Suzhou, 215031 Jiangsu China

**Keywords:** Traumatic brain injury (TBI), Neuroinflammation, Fetuin-A, Microglia, Necroptosis, Oxidative stress, Nrf-2/HO-1 pathway

## Abstract

**Background:**

The microglia-mediated inflammatory response is a vital mechanism of secondary damage following traumatic brain injury (TBI), but the underlying mechanism of microglial activation is unclear.

**Methods:**

Controlled cortical impact (CCI) was induced in adult male C57BL/6J mice, and glutamate was used to construct a classical in vitro injury model in the primary microglia. Microglial activation was determined by western blot and immunostaining. The inflammatory factors were measured by enzyme-linked immunosorbent assay. The oxidative stress marker and mitochondrial reactive oxygen species (ROS) were measured by immunoblotting and MitoSox Red staining. Transmission electron microscopy was used to observe the typical morphology of necroptotic cells.

**Results:**

Our quantitative proteomics identified 2499 proteins; 157 were significantly differentially expressed in brain tissue between the 6 h after CCI (CCI6h) group and sham group, and 109 were significantly differentially expressed between the CCI24h and sham groups. Moreover, compared with the sham group, the terms “acute-phase response”, “inflammation”, and “protein binding” were significantly enriched in CCI groups. Fetuin-A, a liver-secreted acute-phase glycoprotein, was involved in these biological processes. Using an experimental TBI model, we found that the Fetuin-A level peaked at 6 h and then decreased gradually. Importantly, we showed that administration of Fetuin-A reduced the cortical lesion volume and edema area and inhibited the inflammatory response, which was associated with suppressing microglial necroptosis, thus decreasing microglial activation. Furthermore, administration of Fetuin-A attenuated mitochondrial oxidative stress in glutamate-treated microglial cells, which is a critical mechanism of necroptosis suppression. In addition, we demonstrated that Fetuin-A treatment promoted translocation of nuclear factor erythroid 2-related factor 2 (Nrf-2) from the cytoplasm to the nucleus in vivo; however, the Nrf-2 inhibitor ML385 and si-heme oxygenase-1 (si-HO-1) disrupted the regulation of oxidative stress by Fetuin-A and induced increased ROS levels and necroptosis in glutamate-treated microglial cells. Fetuin-A also protected neurons from adverse factors in vivo and in vitro.

**Conclusions:**

Our results demonstrated that Fetuin-A activated Nrf-2/HO-1, suppressed oxidative stress and necroptosis levels, and thereby attenuates the abnormal inflammatory response following TBI. The findings suggest a potential therapeutic strategy for TBI treatment.

**Supplementary Information:**

The online version contains supplementary material available at 10.1186/s12974-022-02633-5.

## Background

Traumatic brain injury (TBI) is a common neurological disease worldwide that is commonly caused by fights, traffic accidents, and playing sports and leads to high mortality and disability rates [1, 2]. The development of TBI is generally divided into two stages. Surgical treatment is the first choice for mechanical injury in the first stage [3, 4]. However, the pathophysiological process of the second stage is complicated and includes a pro-inflammatory response, oxidative stress, local hypoxia/reoxygenation, and accumulation of neurotoxic substances [5, 6]. Our previous study demonstrated that an abnormal inflammatory response leads to neuronal death [7]. As the intrinsic immune effector cells of the central nervous system (CNS), microglia are activated rapidly, mobilize to the injured area to clear debris, and initiate a variety of sterile immune reactions [8]. Therefore, microglial activation serves as the innate immune response in the CNS, which defense against injury [9, 10]. However, uncontrolled and excessive microglial activation can become detrimental by inducing neurological dysfunction and neurodegeneration [11]. Thus, inhibition of abnormal microglial activation is widely considered as a novel therapeutic strategy to improve the prognosis of TBI patients.

Different types of cell death have been found to occur during brain trauma, such as apoptosis, necroptosis, and ferroptosis [12–14]. Necroptosis is initiated by activation of tumor necrosis factor alpha (TNFα) and/or Fas, which differentiates this process from caspase-dependent apoptosis [15]. Activated receptor-interacting protein 1 (RIPK1) and receptor-interacting protein 3 (RIPK3) combine to form a necrosome complex, which is considered a pivotal regulator of necroptosis. Finally, the activated mixed lineage kinase domain-like pseudokinase (MLKL), which is phosphorylated by the necrosome, binds to cell membrane phospholipids and causes the membrane to rupture and release large amounts of inflammatory factors [16–18], which is consistent with the pathological process caused by abnormal activation of microglia [19]. Recent findings have shown that abnormal oxidative stress in mitochondria leads to necroptosis [20]. However, the relationship between oxidative stress and necroptosis in TBI, particularly in the inflammatory response, has rarely been investigated. Nuclear factor erythroid 2-related factor 2 (Nrf-2) is a major member of the endogenous antioxidant system, which inhibits oxidative stress and increases the levels of other endogenous genes such as heme oxygenase-1 (HO-1) in numerous pathological processes [21, 22]. Thus, these studies indicate that attenuation of oxidative stress and activation of the Nrf-2/HO-1 signaling pathway may be involved in necroptosis following TBI.

Proteomics is an experimental method for qualitative or quantitative analysis of proteins in complex samples through a variety of techniques and has been widely used in studies of various human diseases, such as gastric cancer and hepatocellular carcinoma [23, 24]. In this study, we identified all proteins in the peri-contusional area of mice following controlled cortical impact (CCI) using label-free liquid chromatography–mass spectrometry (LC–MS) proteomic analysis and identified differentially expressed proteins by comparison with sham brain tissues.

Pedersen first isolated Fetuin-A from bovine fetal serum in 1944 [25]. In the subsequent 77 years, the multifunctional properties of Fetuin-A have been clarified [26–28]. Fetuin-A is a glycoprotein that is mainly synthesized in hepatocytes and secreted into the blood circulation [28]. Previous studies on Fetuin-A have mostly focused on the vasculature and metabolism, and Fetuin-A has been found to regulate normal bone mineralization, inhibit the insulin signaling pathway, and promote adipocyte dysfunction [27, 29]. Interestingly, recent studies have shown that high plasma Fetuin-A levels predict an increased risk of ischemic stroke [30]. Moreover, peripheral administration of Fetuin-A has been reported to attenuate the inflammatory response in early cerebral ischemic injury [31]. However, the role of Fetuin-A in TBI has not been examined.

In this study, we investigated the role and mechanisms of Fetuin-A in the abnormal inflammatory response following TBI. We verified that Fetuin-A suppressed reactive oxygen species (ROS) production in microglial cells via upregulation of the Nrf-2 level in the nucleus, which promotes HO-1 transcription, thus alleviating necroptosis and the abnormal inflammatory response. Our study shows that a Fetuin-A-driven cascade inhibits inflammation, which can be a potential therapeutic strategy for TBI patients.

## Materials and methods

### Patients

Human brain tissues, including three TBI tissues and three non-contusive tissues (control), were obtained from the Department of Neurosurgery at the First Affiliated Hospital of Nanjing Medical University, which was approved by the Institutional Review Board. Detailed information of the brain tissues is displayed in Table 1. Brain tissues resected from patients were snap-frozen and stored in liquid nitrogen until assay. The Ethics Committee of Nanjing Medical University approved the use of human brain tissue, and all procedures were conducted in accordance with approved guidelines. The participant’s explicit permission was obtained, and the patient provided informed consent.

**Table 1:** Clinical information of human brain specimens

No. of patient	Gender	Age	Diagnosis	Time to injury	Site
Ctrl					
1	Male	39	Epilepsy	–	Left temporal lobe
2	Female	63	Arteriovenous malformation	–	Left temporal lobe
3	Male	62	Arterial aneurysm	–	Right frontal lobe
TBI					
1	Female	35	TBI	24 h	Left frontal lobe
2	Male	58	TBI	48 h	Right frontal lobe
3	Female	53	TBI	52 h	Right frontal lobe

### Animals and experimental design

The Laboratory Animal Center of Nanjing Medical University provides adult male C57BL/6J mice (25 ± 2 g). All animals were kept in an SPF condition with regulated temperature (22 ± 2 °C), a light and dark cycle of 12:12 h and received standard laboratory animal food and water. The Institutional Animal Care and Use Committee of Nanjing Medical University approved all research protocols and animal experiments in accordance with the guidelines of the Animal Care and Use Committee (National Institutes of Health Publication No. 85-23, revised 1996).

Six separated experiments were performed as follows.

#### Experiment 1

To understand the changes of proteins content in brain after CCI. 32 C57BL/6J mice were randomly divided into three groups: sham (*n* = 16), 6 h (*n* = 8), 24 h (*n* = 8) after CCI induction. We collected the cerebral cortex around the lesion at 6 h or 24 h after CCI and then we identify global differences in protein expression between CCI and sham mouse groups by proteomic analysis (Additional file 1: Fig. S1A).

#### Experiment 2

To determine the endogenous Fetuin-A at each time point after CCI, 30 C57BL/6J mice were randomly divided into five groups: 0 (sham), 6 h, 12 h, 24 h, 72 h (*n* = 6/group) after CCI induction. The mice were all killed at the scheduled time point and the brain tissue was collected for western blot analysis and immunofluorescence (IF) assays to measure Fetuin-A expression (Additional file 1: Fig. S1B).

#### Experiment 3

To investigate the intravenous administration of Fetuin-A in the post-CCI brain injury. 48 C57BL/6J mice were randomly assigned into five groups: sham, sham + FITC-Labeled Fetuin-A (25, 50, 75 mg/kg), CCI, CCI + FITC-Labeled Fetuin-A (25, 50, 75 mg/kg) (*n* = 6/group). The FITC-labeled Fetuin-A was administered by tail vein injection 15 min following CCI. Tissue samples were collected at 6 h or 3 w after CCI, and then we detect the content of Fetuin-A by western blot analysis, immunofluorescence assays and immunohistochemistry (ICH) assays (Additional file 1: Fig. S1C, D). Besides, we test whether intravenous administration of Fetuin-A has biological toxicity via H&E and TUNEL staining (Additional file 1: Fig. S1E).

#### Experiment 4

To explore the damage degree and repair of blood brain barrier (BBB) after CCI, 30 C57BL/6J mice were randomly assigned into five groups: 0 (sham), 1 d, 3 h, 7 d, 3 w (*n* = 6/group) after CCI induction. The mice were all killed at the scheduled time point and the brain tissue was collected for western blot analysis and immunofluorescence assays to measure cortex BBB dysfunction (Additional file 1: Fig. S1F).

#### Experiment 5

To detect the role of Fetuin-A in CCI model and explore the underlying mechanism of Fetuin-A, 70 C57BL/6J mice were randomly assigned into four groups: sham, CCI, CCI + Fetuin-A (50 mg/kg), CCI + sh-Ctrl, CCI + sh-Fetuin-A-1, CCI + sh-Fetuin-A-2, CCI + sh-Fetuin-A-3 (*n* = 10/group). Tissue samples were collected at 6 h or 3 w after CCI, and then we detect the changes of tissue lesion, cell death, microglia activation and neutrophil infiltration via H&E, TUNEL staining, Brain water content, lesion volume analysis, western blot analysis, immunofluorescence assays and immunohistochemistry assays (Additional file 1: Fig. S1G, H).

#### Experiment 6

To investigate whether the therapeutic effect of Fetuin-A was dependent on microglia following CCI, 36 C57BL/6J mice were randomly assigned into six groups: sham, sham + vehicle (Veh), sham + PLX5622 (PLX), CCI + Veh, CCI + PLX, CCI + PLX + Fetuin-A (*n* = 6/group). Tissue samples were collected at 6 h or 3 w after CCI, and we detect the depletion efficiency of microglia via immunohistochemistry assays. Besides, we explored the tissue lesion, neuron death via H&E, TUNEL staining, Brain water content, lesion volume analysis (Additional file 1: Fig. S1I).

### CCI model

As described previously, 8-week-old mice were subjected surgery to produce a controlled cortical impact (CCI) model [32]. Mice were anesthetized with 4% isoflurane in 70% nitrous oxide and 30% oxygen, and maintained with 1.5% isoflurane. The body temperature was maintained at 37 ± 0.5 °C by a heating blanket. Then, we performed a 4-mm-diameter craniotomy in the left parietal bone (the relative coordinates centre of craniotomy to bregma: 1.5 mm posterior and 2.5 mm lateral). For the sham groups, only the dura mater was exposed. In the TBI groups, the exposed dura mater was struck by impactor at 6.0 ± 0.2 m/s velocity with 1.4 mm depth and 50-ms dwell time. After the injury, we closed the skin incision, and then mice were caged.

### Recombinant adenovirus administration

The recombinant adenoviruses sh-Fetuin-A (AD-shFetuin-A) and control (AD-shcontrol) were constructed by GenePharma (Shanghai, China). The three shRNA targeting sequences were as follows: (1) sense: 5′-CCGUGGACUACCUCAAUAATT-3′, antisense: 5′-UUAUUGAGGUAGUCCACGGTT-3’; (2) sense: 5′-GGGAGAAACUCUUCAUUCUTT-3′, antisense: 5′-AGAAUGAAGAGUUUCUCCCTT-3′; (3) sense: 5′-GCCUUCAACACACAGAAUATT-3′, antisense: 5′-UAUUCUGUGUGUUGAAGGCTT-3′. The recombinant adenoviruses were produced and purified according to the manufacturer's instructions. The adenoviruses were subjected to large-scale amplification in AD293A cells and were subsequently collected from supernatant, condensed, and purified. The titers of adenoviruses were determined and calibrated in 293T cells. For tail intravenous injection, C57BL/6J mice were injected with 1 × 10^10^ viral particles in a total volume 200 μL. Seven days were needed for successful transfection before CCI treatment.

### Brain tissue preparation

**Fig. 1:**
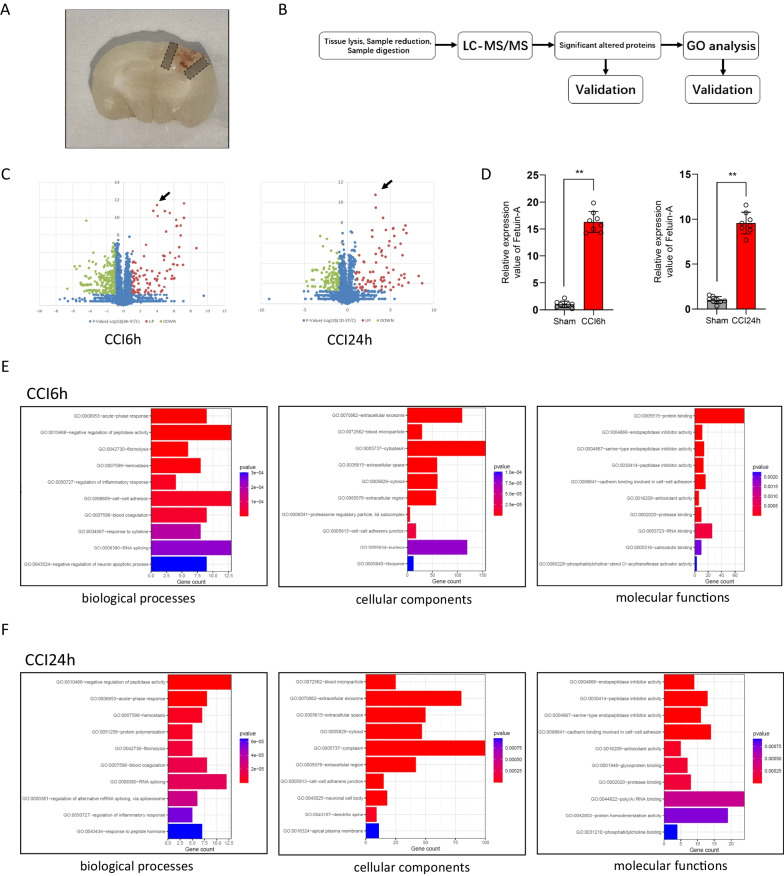
Protein identification and quantification by label-free LC–MS/MS. Quantitative proteomics analysis of the sham mice and TBI mice, and validation of differentially expressed Fetuin-A in different groups. And the animals in the sham group underwent all surgical procedures for TBI induction except the traumatic step. **A** Location of collected tissues was labeled. Collected cortical tissues were marked by gray frame. **B** Experimental design for proteomic analysis in the mice brain tissues by label-free LC–MS. **C** Volcano plot graph of 2499 nonredundant proteins. The − log10 (*P*-value) was plotted against the log2 (ratio TBI/Sham). The upregulated proteins in TBI tissues were marked with red dots (black arrow: Fetuin-A), and the downregulated proteins in TBI tissues with green dots. Blue plots represented the rest of genes with no significant expression change. **D** The relative protein level of Fetuin-A in CCI (*n* = 8 samples) vs. sham (*n* = 8 samples). **E**, **F** Classification of proteins identified through proteomics into their molecular biological processes (BP), cellular components (CC), and molecular functions (MF). The top 10 Gene Ontology (GO) enrichment analysis were listed. Data are presented as the means ± SD; **P < 0.01 vs. sham group

To prepare brain tissue, mice from each group were randomly selected after experimental TBI. The mice were anesthetized with 10% chloral hydrate (0.4 mL/100 g) injected intraperitoneally. After successful anesthesia, the mice were placed on a wooden board in the supine position. The sternum was cut, the xiphoid was lifted and the chest cavity was cut. Then, the right atrial appendage was cut, and the perfusion needle was inserted from the apex of the left ventricle. A pre-cooled phosphate-buffered saline (PBS) solution was perfused into the lungs and liver, the color of which became grayish-white. Some animals were then reperfused with 4% paraformaldehyde according to the type of experiment. Finally, the mouse was decapitated, the skull was exposed and separated, and the brain was gently removed. Cerebral cortex tissue around the lesion (Fig. 1A) was collected and snap-frozen on dry ice, then stored at − 80 °C for liquid chromatography–mass spectrometry (LC–MS) analysis and western blot assays. For immunostaining assays and hematoxylin and eosin (H&E) staining, the brain tissue was fixed in neutral formalin solution for 24 h, then dehydrated by alcohol gradients, and finally embedded in paraffin.

### LC–MS analysis and proteomic data processing

All the 16 pairs of experimental samples were performed using a QExactive Plus Orbitrap™ mass spectrometer (Thermo Fisher Scientific, Waltham, MA, USA) equipped with a nano-electrospray ion source as described previously [23]. Samples were dissolved in water/formic acid (0.1%, v/v), and peptides were separated by reversed phase liquid chromatography using an EASY-nLC™ 1000 system (Thermo Fisher Scientific). A set-up of pre-column and analytical column was used. The pre-column was a 2 cm EASY-column (1D 100 μm, 5 μm C18) (Thermo Fisher Scientific) while the analytical column was a 10-cm EASY-column (ID 75 μm, 3 μm, C18) (Thermo Fisher Scientific). Peptides were washed with a 90 min linear gradient from 4 to 100% acetonitrile at 250 nL/min. The mass spectrometer was operated in positive ion mode, acquiring a survey mass spectrum with resolving power 70,000 and consecutive high collision dissociation fragmentation spectra of the 10 most abundant ions. The acquired data (.RAW-files) were processed by Maxquant (Version 1.5.0.1) against the Uniprot-Swissprot database using an extracted FASTA file specified for “mouse” taxonomy. The search parameters included: maximum 10 ppm and 0.02 Da error tolerance for the survey scan and MS/MS analysis; enzyme specificity was trypsin; maximum 2 missed cleavage sites allowed; cysteine carbamidomethylation was set as static modification; oxidation (M) was set as variable modifications. The protein identification was based on 95% confidence per protein. The acquired data were subject to Gene Ontology and protein class analysis via the PANTHER (http://pantherdb.org/).

### Drug administration

Recombinant Mouse Fetuin-A was provided by R&D Systems Inc (USA) and labeled with fluorescein isothiocyanate (FITC) by using the EZ-Label FITC Protein Labeling kit (Pierce Biotechnology, Rockford, IL, USA) according to the manufacturer’s instructions. At 15 min following CCI, the FITC-labeled Fetuin-A (25, 50, 75 mg/kg) was administered through the tail vein. The presence of FITC-labeled Fetuin-A was examined by a fluorescence microscope.

Microglial cells were treated with Recombinant Mouse Fetuin-A (R&D Systems, USA) at different physiologically relevant concentrations that ranged from 150–900 μg/mL [33].

PLX5622, which is a colony-stimulating factor 1 receptor (CSF1R) inhibitor, was provided by Plexxikon Inc. (Berkeley, CA) and formulated in AIN-76A standard rodent chow by Research Diets Inc. (New Brunswick, NJ) at a concentration of 1200 parts per million. The mice were fed the PLX5622 diet to deplete microglia or AIN-76A chow as vehicle control. After being raised for 2 weeks, mice were subjected to CCI injury for further experiments.

*N*-Acetyl-l-cysteine (NAC) was purchased from Sigma Chemicals (USA). Microglial cells were treated with NAC (10 mM) 1 h before stimulation with glutamate for 24 h.

ML385, which is Nrf-2 inhibitor, was purchased from Selleck Chem. Microglial cells were treated with different dilution concentrations (0, 2, 4, 6, 8, 10 μmol/L) of ML385 for 72 h.

### Extraction of primary neuron and microglia and cell culture

Primary cortical neurons were obtained from the cerebral cortices of 1-day-old C57BL/6J mice. The mice were decollated, and the intact brain was immediately immersed in pre-cooled Dulbecco’s modified Eagle’s medium/nutrient mixture F-12 (DMEM/F12) medium. Then the cerebral cortex was dissected and digested with 0.25% trypsin and DNase at 37 °C for 20 min (with shaking every 5 min). The digestion was terminated by the addition of horse serum and was then filtered through the cell strainer. Then, the filtrate was centrifuged at 1000 rpm for 5 min, and the supernatant was discarded. We resuspended the cell pellet in DMEM/F12 medium containing 10% horse serum and 2% penicillin and streptomycin (Gibco). The 6- or 12-well culture dishes contained 6–7 × 100,000 cells per well. After about 4 h, the medium was replaced with neurobasal medium containing 2% B27 and 0.5 mM glutamate, and the dishes were placed in a cell incubator at 37 °C, with 5% CO_2_.

The preparation of primary microglia was similar to that of neurons. The difference was that DMEM/F12 containing 10% fetal bovine serum, 1 mM sodium pyruvate, 2 mM l-glutamine, 100 mM nonessential amino acids, 50 U/mL penicillin, and 50 mg/mL streptomycin (all from Gibco) was the medium used for the cells. After plating, cells were cultured for 2 days in a 150-cm^2^ culture flask pretreated with poly-d-lysine (Sigma, USA). Within 2 weeks of seeding, glial cells formed a confluent monolayer. Microglia were separated from astrocytes by shaking the flask and were collected by centrifuging.

### Cell transfection and in vitro injury model

The siRNA targeting HO-1 was purchased from GenePharma (Shanghai, China), and the sequences of siRNAs were as follows: sense: (1) sense: 5′-CCACACAGCACUAUGUAAATT-3′, antisense: 5′-UUUACAUAGUGCUGUGUGGTT-3’; (2) sense: 5′-CUCGAAUGAACACUCUGGATT-3′, antisense: 5′-UCCAGAGUGUUCAUUCGAGTT-3′; (3) sense: 5′-CUGCUCAACAUUGAGCUGUTT-3′, antisense: 5′-ACAGCUCAAUGUUGAGCAGTT-3′. At 6 h after transfection, the medium was changed to DMEM with 10% fetal bovine serum. Then, 24 h later, the primary microglia were treated with 100 μm glutamate (Glu) to induce cellular injury for 24 h according to the study protocol.

### Western blotting

Proteins were extracted from tissues or cells following the previous description [34]. The proteins were separated by 10% or 12% SDS-PAGE gel and then placed to polyvinylidene fluoride (PVDF) membranes (Merck Millipore). Membranes were blocked in 5% non-fat dried milk for 2 h at ambient temperatures and then incubated overnight at 4 °C with antibodies against GAPDH (1:2000, #5174; Cell Signaling Technology), Fetuin-A (1 µg/mL, ab112528; Abcam), Fetuin-A (1:2000, ab187051; Abcam), Iba-1 (1:1000, ab178846; Abcam), CD16 (1:1000, ab223200; Abcam), Cleaved Caspase-3 (1:1000, #9661; Cell Signaling Technology), Bax (1:1000, ab32503; Abcam), Bcl-2 (1:2000, ab182858; Abcam), RIPK3 (1:1000, #DF10141; Affinity), MLKL (1:1000, DF7412; Affinity), p-RIP3 (1:1000, #91702; Cell Signaling Technology), p-MLKL (1:1000, #37333, Cell Signaling Technology), Nrf-2 (1:1000, #12721, Cell Signaling Technology), Histone H3 (1:2000, ab1791; Abcam) and HO-1 (1:1000, #43966; Cell Signaling Technology) followed by incubation with horseradish peroxidase-conjugated secondary antibody (Beyotime, China, A0208, A0216, 1:5000) for 2 h. After washing with PBST, we ascertained the protein bands by using SuperSignal^®^ Maximum Sensitivity Substrate (Thermo Fisher Scientific). And we used ImageJ software (National Institutes of Health) to calculate the achieved bands’ optical density. Samples derived from the same experiment and blots are processed in parallel. The source data file contains uncropped and unprocessed scans of blots.

### Immunostaining and hematoxylin and eosin (H&E) assay

For immunofluorescence assays, the 8-µm-thick frozen brain sections were permeabilized with 0.1% Triton X-100 (Sigma-Aldrich, St Louis, MO; USA, X100) for 15 min, and blocked with 5% normal goat serum (Millipore; S26-LITER) at 37 °C for 1 h. Then, the sections were incubated with primary antibodies at 4 °C throughout the night, washed three times with PBS, and incubated with Alexa Fluor 488- or CyTM3-conjugated secondary antibodies (Jackson, USA, 1:500) for 2 h at ambient temperature. After additional washing three times with PBS, nuclei underwent staining process using Hoechst (C1018, Beyotime, China) at ambient temperature for 10 min. For immunofluorescence staining of cells, different methods were used. The primary microglial and neuronal cells were plated on glass slides which precoated with poly-lysine (PLL). Then, the cells were fixed by 4% paraformaldehyde (PFA) for 1 h. The rest of the steps were the same as the immunofluorescence assay of brain sections. Finally, a laser scanning confocal microscope (TCS SP5II, Leica, Wetzlar, Germany) was used to observe immunoreactivity, and each section was imaged randomly by scanning 3–6 fields in each quadrant. Quantitative analysis of signal intensities was performed manually by a blinded investigator using ImageJ. For the count of positive cells, the blinded investigator selected relevant areas and counted them manually. And the number of positive staining cells with a signal-to-noise ratio (S/N) ≥ 10.0 was quantified to distinguish positive fluorescence intensity (The Signal) from spontaneous fluorescence (The Noise) [35].

For immunohistochemistry assays, 8-µm-thick brain sections were incubated overnight with the primary antibodies at 4 °C. And the sections underwent incubation with the secondary antibody for 30 min at ambient temperature, and washed with PBS, and incubated with DAB for 15 min at 37 °C. The sections were imaged by a light microscope (Leica), and each section was imaged randomly by scanning 3–6 fields in each quadrant. The average absorbance was measured by ImageJ, and the proportion of positive cells was counted manually.

Hematoxylin and eosin (H&E) staining was performed on prepared 8-mm-thick sections. Prepared 8-mm-thick sections. Slides were imaged under a light microscope (Leica).

The following primary antibodies were used to perform immunostaining: Fetuin-A (1 µg/mL, ab47979; Abcam), Fetuin-A (1:2000, ab187051; Abcam), Iba-1 (1:500, ab178846; Abcam), CD16/32 (1:500, ab223200; Abcam), MPO (1:1000, ab208670; Abcam), p-RIP3 (1:400, #91702; Cell Signaling Technology), p-MLKL (1:1600, #37333, Cell Signaling Technology), Nrf-2 (1:400, #12721, Cell Signaling Technology).

### Terminal deoxynucleotidyl transferase mediated dUTP nick end labeling (TUNEL) assay

According to the manufacturer’s instructions, a TUNEL assay (C1089, Beyotime, China) was used to detect cell death. Briefly, 12-μm brain sections or cells were fixed in 4% PFA. And then they were incubated with 50 μL TUNEL reaction mixture and 0.3% Triton X-100 in the dark (37 °C) for 1 h. After rinsing three times with PBS, DAPI was used to visualize cell nuclei. Images were obtained using a Nikon Eclipse E600 microscope (Nikon, Melville, NY).

### Brain water content and cortical lesion volume

The mice brain was removed immediately after decapitation and weighed. Subsequently, the brain was dried at 70 °C for 72 h, and the dry weight determined. The brain water content was obtained based on water content (%) = [(wet weight − dry weight)/wet weight] × 100% [36].

A slice thickness of 0.5 mm was used for lesion volume measurement. Lesion volume was assessed from the summation of areas of defect on each slice and multiplied by slice thickness. ImageJ was used to quantitatively analyze the data.

### Enzyme-linked immunosorbent assay (ELISA) assay

The brain tissues were collected from the mice after CCI 6 h. Briefly, added 5 mg of brain tissue to a tube along with 300 µL of extraction buffer and mix for 2 h at 4 °C with an electric homogenizer. Then, the brain tissues were centrifuged at 13,000 rpm for 20 min at 4 °C. The supernatant was extracted and diluted with sample buffer at a ratio of 1:1 and loaded onto the wells in duplicates. TNF-α, IL-6, IL-1β, and IL-10 expressions in the brain tissues were measured using a specific enzyme-linked immunosorbent assay (ELISA; R&D Systems Inc) according to the manufacturer’s instruction.

The conditioned medium was moved to a centrifuge tube and centrifuged at 1500 rpm for 10 min at 4 °C and samples were loaded onto the wells in duplicates. TNF-α, IL-6, IL-1β, and IL-10 expressions in culture supernatants were measured by a specific ELISA according to the manufacturer’s instruction.

### Cell viability and lactate dehydrogenase (LDH) assay

We ascertained cell viability by Cell Counting Kit-8 (CCK-8, CK04, Dojindo, Tokyo, Japan) assay. In brief, cells was cultured in a 96-well plate, and incubated with the reagent at 37 °C for 2 h. Then, optical density (OD) values were measured at 450 nm by a Thermo Multiskan FC microplate photometer.

Cellular injury-induced cytotoxicity was measured by Cytotoxicity Detection Kit (C0017, Beyotime Biotech, China) in line with the directions of the manufacturer.

### Transmission electron microscopy

The microglial cells were fixed in PBS (pH 7.4) containing 2.5% glutaraldehyde for at least 1 h at room temperature. After this step, cells were post-fixed with 1.5% osmium tetroxide for 2 h at 4 °C and dehydrated with ethanol, followed by embedding in epoxy resin. The ultrastructure of the microglial cells (70 nm ultrathin sections) was observed by transmission electron microscope (Quanta 10, FEI Co.)

### JC-1 fluorescence assay

The mitochondrial membrane potential was measured using JC-1 (C2003S, Beyotime, China) fluorescence mitochondrial imaging. The microglial cells were incubated with JC-1 solution for 20 min at 37 °C. And then, the cells were rinsed twice using JC-1 buffer. Images were obtained using a Nikon Eclipse E600 microscope (Nikon, Melville, NY). The ratio of red to green fluorescence represented the mitochondrial membrane potential.

### Extraction of cytoplasmic and nuclear protein

After the different treatment described above, Nuclear and Cytoplasmic Protein Extraction Kit (P0027, Beyotime Biotech, China) was used to separate cytoplasmic protein and nuclear protein, and the variation of Nrf-2 expression was detected by Western blot assessment.

### Measurement of mitoROS levels

After culture of microglial cells in 6-well dishes with 6 × 10^4^ cells/well, the mitoROS was examined by MitoSOX molecular probes (Invitrogen, CA) according to the manufacturer’s instruction. At the end of treatment, Nikon Eclipse E600 microscope (Nikon, Melville, NY) was used to obtain the image of MitoROS at λ579 nm.

### Determination of malondialdehyde (MDA), GSH and GSSH level

The MDA, GSH and GSSH level was measured with the Lipid Peroxidation MDA Assay Kit (S0131S, Beyotime Biotech, China) and GSH and GSSG Assay Kit (S0053, Beyotime Biotech, China). After the different treatment described above, the cells were washed with PBS (pH 7.4) and were lysed subsequently. The lysates were centrifuged at 12,000 rpm for 10 min at 4 ℃. Then, the supernatant was collected and the absorbance at 532 nm was measured by a microplate reader (Biotech, Winooski, VT, USA). Finally, the datum was normalized by the protein concentration in each sample.

### Co-immunoprecipitation (Co-IP) assay

Co-IP was performed following the previous description [37]. In brief, the microglial cells were lysed and total lysates were harvested by weak RIPA lysis buffer (Cell Signaling Technology, Danvers, MA, USA). After clearing with 50% protein A/G agarose for 1 h, the 500 mL of extracted proteins were incubated with primary antibodies of corresponding dilution overnight at 4 °C. Then, The immune complexes were pulled down with protein A/G agarose in a shaker at 4 °C for 4 h. Microbeads were collected and washed, and then proteins were eluted through boiling in 1× loading buffer followed by immunoblotting analysis.

### Statistical analysis

All data were analyzed with GraphPad 8.0 software and expressed as the mean ± standard deviation (SD) of at least three independent experiments. Gray level and fluorescence intensity were detected with ImageJ. An unpaired Student’s *t*-test or one-way analysis of variance (ANOVA) plus Tukey’s post hoc test was applied to compare the differences between two groups. For the difference of groups at the same time point, the data were analyzed using one-way ANOVA followed by Tukey’s post hoc test. Two-way ANOVA was used to compare the data from all groups. *P* < 0.05 was considered as a significant difference.

## Results

### Fetuin-A was overexpressed after brain injury

In this study, label-free LC–MS proteomic analysis was used to identify global differences in protein expression between the CCI and sham mouse groups (Fig. 1B). In total, 2499 nonredundant proteins were identified and quantified using MaxQuant 1.5.0.1. The analysis indicated that 297 proteins (93 upregulated proteins [red plots] and 204 downregulated proteins [green plots]) were significantly differentially expressed between the CCI6h group (*n* = 8) and sham group (*n* = 8) based on the log2 (fold change) values. Additionally, 187 proteins (78 upregulated proteins [red plots] and 109 downregulated proteins [green plots] were significantly differentially expressed between the CCI24h group (*n* = 8) and sham group. The volcano plot was generated based on the differential expression ratios and *P* values (Fig. 1C). The level of Fetuin-A was significantly increased in the CCI6h and CCI24h groups compared with that in the sham group (Fig. 1D). Given that the progression of TBI was accompanied by Fetuin-A upregulation, we further investigated its role in craniocerebral injury. Next, we performed Gene Ontology (GO) analysis of the proteins that changed significantly following CCI and identified the Top10 gene enrichment regions for “biological processes”, “cellular components”, and “molecular functions”, respectively. Interestingly, the terms of “acute-phase response”, “inflammation”, and “protein binding” were significantly enriched in the CCI groups compared with the sham group (Fig. 1E, F), and Fetuin-A has been shown to be involved in these biological processes. Considering the involvement of Fetuin-A in the inflammatory regulation of multiple diseases reported in previous studies [31], these results suggested that Fetuin-A may be associated with the pathophysiological processes following CCI, but the specific mechanism still needs to be explored.

**Fig. 2:**
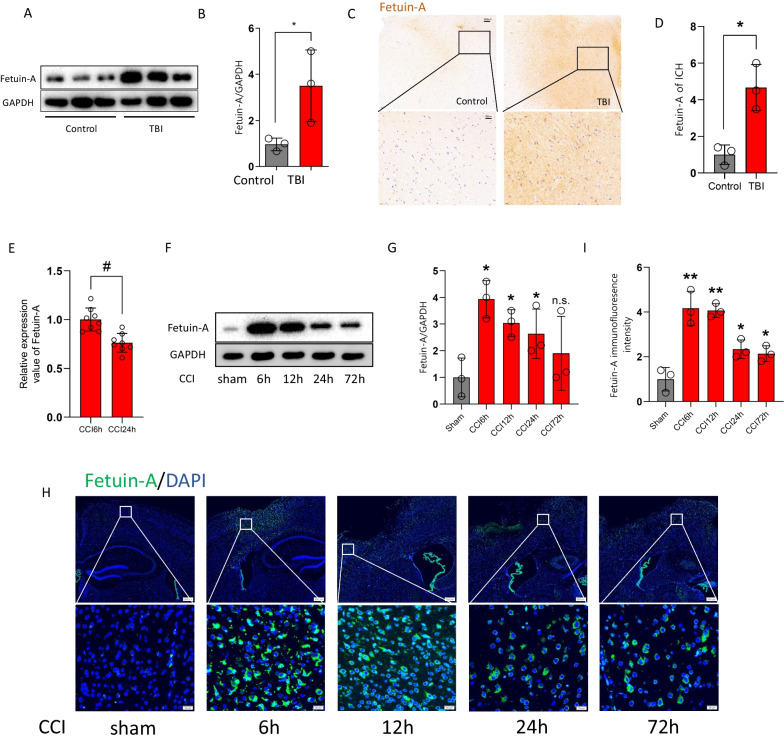
Fetuin-A is overexpressed after brain injury. **A**, **B** Western blot analysis and densitometric quantification of Fetuin-A expression by ImageJ in brain tissue of control (*n* = 3 samples) and TBI (*n* = 3 samples) patients. **C**, **D** Immunohistochemistry assessment of Fetuin-A expression in brain tissue from control and TBI patients. Scale bar is 20 or 100 μm. The relative intensity of Fetuin-A was detected with Image-J software. **E** The relative protein level of Fetuin-A in CCI6h vs. CCI24h (*n* = 8 samples) from quantitative proteomics analysis. **F**, **G** Protein levels of Fetuin-A obtained from sham mice and CCI mouse brain tissue. GAPDH is used as the loading control. And bar graphs show the results of analysis (by band density analysis) of Fetuin-A (sham = 3, CCI = 3). **H**, **I** Immunofluorescence assessment of Fetuin-A (green). Scale bar is 20 or 200 μm. The relative immunofluorescence intensity of Fetuin-A was detected with Image-J software. Data presented as mea*n* ± SD (*n* = 5). *P < 0.05 vs. control group or sham group, **P < 0.01 vs. control group or sham group, and ^#^*P* < 0.05 vs. CCI6h group

We used western blot to compare the levels of *FETUIN-A* in severe trauma patients and control patients. The results demonstrated that *FETUIN-A* expression was markedly increased after TBI (Fig. 2A, B). This was similar to the results of immunostaining (Fig. 2C, D). Our proteomic analysis indicated that the content of Fetuin-A in the CCI6h group was higher than CCI24h group (Fig. 2E). And our western blot assessments showed that the content of Fetuin-A peaked at 6 h, and it was maintained at a high level until 24 h following CCI (Fig. 2F, G). In addition, immunofluorescence staining showed that the intensity of the Fetuin-A signal in the CCI6h group was higher than that in the CCI72h group (Fig. 2H, I).

### Peripheral administration of Fetuin-A exerted protective effects after CCI

**Fig. 3:**
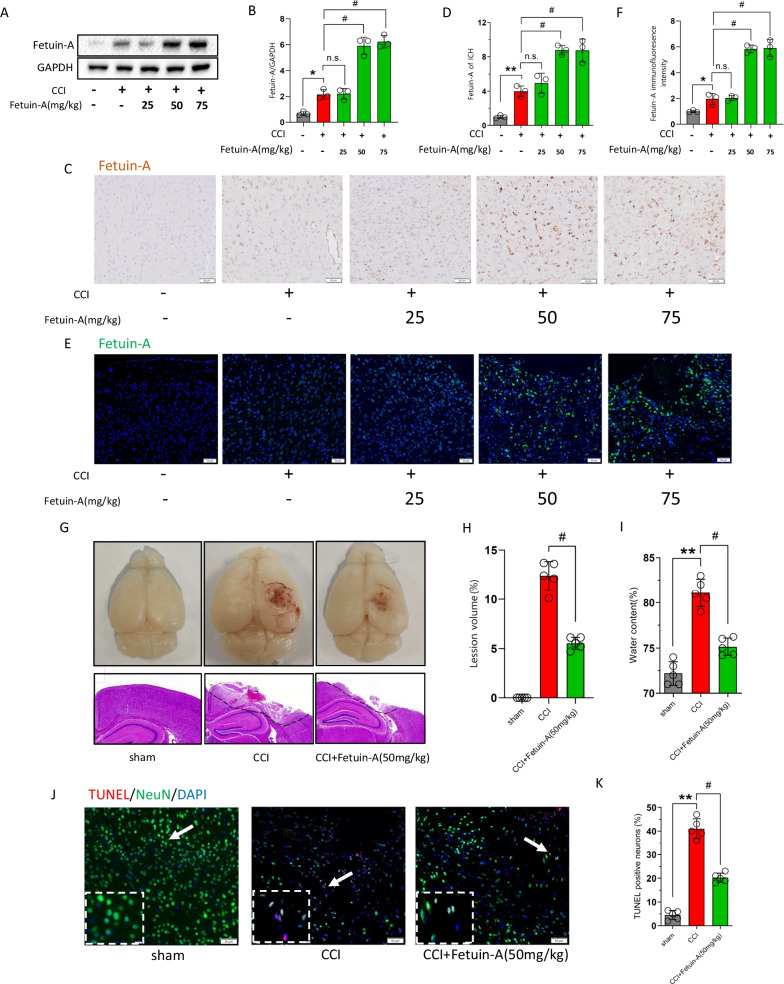
Peripheral administration of Fetuin-A exerted protective effects after CCI. The mice were treated with Fetuin-A at indicated concentrations after CCI. **A**, **B** Protein levels of Fetuin-A obtained from sham mice and CCI mice at 6 h. GAPDH was used as the loading control. And bar graphs show the results of analysis (by band density analysis) of Fetuin-A (sham = 3, CCI = 3). **C**, **D** Immunohistochemistry assessment of Fetuin-A in sham and CCI at 6 h (scale bar = 50 μm, *n* = 3). The relative intensity of Fetuin-A was detected with Image-J software. **E**, **F** Immunofluorescence assessment of Fetuin-A. Scale bar is 50 μm. The relative immunofluorescence intensity of Fetuin-A was detected with Image-J software (*n* = 3). **G** Representative photographs of whole brains and H&E staining of hemicerebrum sections (*n* = 3). **H**, **I** Lesion volume (*n* = 5) and water content% (*n* = 5) were analyzed by statistical. **J**, **K** Neuron death measured by TUNEL staining. Scale bar is 50 μm (*n* = 5). Data are presented as the means ± SD; *P < 0.05 vs. sham group, **P < 0.01 vs. sham group, and ^#^*P* < 0.05 vs. CCI group

Fetuin-A is mainly secreted by hepatocytes and is present physiologically in small amounts in the CNS [30]. We initially examined its ability to cross the blood–brain barrier (BBB). Fluorescein isothiocyanate-labeled Fetuin-A was intravenously injected (25 to 75 mg/kg) at 15 min following CCI [38], and then the Fetuin-A content was detected by western blot analysis at 6 h following CCI. The results showed that peripherally administered Fetuin-A crossed the BBB, and no significant increase in Fetuin-A content was observed in the brains of CCI mice administered 75 mg/kg Fetuin-A compared with those administered 50 mg/kg Fetuin-A (Fig. 3A, B). Immunostaining showed similar results (Fig. 3C–F). Furthermore, tight junction proteins (ZO-1 and occludin) expression was significantly lower in the CCI groups than in the sham group (Additional file 2: Fig. S2A–B). And immunofluorescence co-localization of tight junction proteins (ZO-1 and occludin) and cerebral microvessels (CD31+) suggested disruption of the BBB integrity after CCI (Additional file 2: Fig. S2C, D). These results suggested that the impacted cortex BBB dysfunction persisted for at least 3 weeks after CCI, and that peripherally administered Fetuin-A was able to cross the dysfunctional BBB into the injured cortex.

Next, we assessed the ability of Fetuin-A to cross the intact BBB and its potential toxicity and side effects in the CNS, and the tissues of brain were harvested at 6 h after injected intravenously. In immunoblotting, the total amounts of Fetuin-A were not different between the groups (Additional file 3: Fig. S3A, B). The results of TUNEL staining, photomicrographs of H&E staining and brain water content analysis demonstrated that there were no significant differences between the normal group and the intravenous injection (Fetuin-A, 50 mg/kg) group (Additional file 3: Fig. S3C–F).

To examine the effects of Fetuin-A in craniocerebral injury, we conducted a series of experiments following intravenous injection of Fetuin-A (50 mg/kg). As shown by Fig. 3G, H, the cortical lesion volume and edema area were obvious in the right cortex at 3 weeks following CCI, and treatment with Fetuin-A promoted protection against CCI. Moreover, the brain water content was evidently decreased in the CCI + Fetuin-A group in comparison with that in the CCI group (Fig. 3I). Furthermore, the number of TUNEL-positive cells was significantly decreased in the CCI + Fetuin-A group compared with that in the CCI group (Fig. 3J, K). Next, we silenced Fetuin-A using recombinant adenoviruses containing shRNA aimed at Fetuin-A (AD-shFetuin-A). The efficiencies of three AD-shFetuin-A sequences were measured by immunoblotting. We found the third sequence to be the most efficient and therefore used it in all subsequent experiments (Additional file 4: Fig. 4A, B). The cortical lesion volume was increased in the CCI + shFetuin-A group compared to the CCI + shcontrol (shctrl) group (Additional file 4: Fig. S4C, D). However, there was no statistical difference in CCI-induced water content after Fetuin-A inhibition (Additional file 4: Fig. S4E). Finally, TUNEL assays indicated that the Ad-shFetuin-A group had an increased number of TUNEL-positive cells compared with that in the AD-shctrl group following CCI (Additional file 4: Fig. S4F, G). Together, these results suggested that intravenous administration of Fetuin-A reduced cell death and exerted protective effects against TBI, whereas silencing Fetuin-A aggravated the damage caused by CCI.

**Fig. 4:**
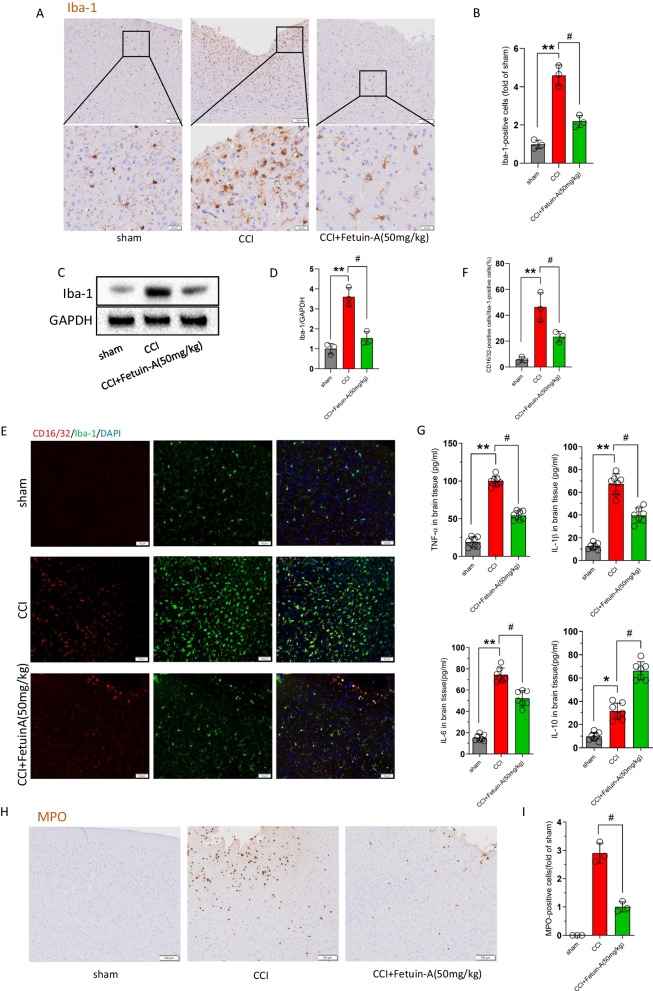
Fetuin-A treatment inhibits microglial activation and ameliorates inflammatory response after CCI. The mice were treated with Fetuin-A at indicated concentrations after TBI. **A**, **B** Immunohistochemistry assessment of Iba-1 in sham or CCI at 6 h and statistical analysis of Iba-1. Reduced process length, branch endpoint and swollen soma were observed in CCI-induced microglial cells (scale bar = 50 μm, *n* = 3). **C**, **D** Western blot analysis of Iba-1 expression from peri-contusional area. GAPDH was used as the loading control. And bar graphs show the results of analysis (by band density analysis) of these proteins (*n* = 3). **E**, **F** The co-localization of CD16/32 with Iba-1 by immunofluorescence assay with representative imaging and statistical analysis. Scale bar is 50 μm (*n* = 3). **G** IL-6, IL-1β, TNF-α, and IL-10 secretion was measured using ELISA (*n* = 7). **H**, **I** Immunofluorescence for MPO in the peri-contusional area and statistical analysis of MPO (scale bar is 100 μm, *n* = 3). Data are presented as the means ± SD; *P < 0.05 vs. sham group, **P < 0.01 vs. sham group, ^#^*P* < 0.05 vs. CCI group

### Fetuin-A treatment inhibited microglial activation and neutrophil infiltration and ameliorated the inflammatory response following CCI

Microglia activation after a TBI is critical to the development of neuroinflammation. To investigate the potential effect of Fetuin-A (50 mg/kg) in microglial activation following craniocerebral injury, we performed immunohistochemistry for the microglial marker Iba-1. Immunohistochemistry indicated that microglia in the CCI group displayed morphological features of activation, including reduced process length, branch endpoint and swollen soma [39]. In addition, the number of Iba-1-positive cells was also significantly increased in the CCI group compared with that in the sham group. However, morphological changes and the increased number of Iba-1-positive cells are alleviated by Fetuin-A administration following CCI (Fig. 4A, B). Consistently, the results of western blotting also indicated that treatment with Fetuin-A attenuated Iba-1 expression induced by CCI (Fig. 4C, D). Furthermore, we double-stained Iba-1 with CD16 to label pro-inflammatory microglia, and as expected, pro-inflammatory microglia were increased at 6 h following CCI and were downregulated by Fetuin-A supplementation (Fig. 4E, F). And the results of enzyme-linked immunosorbent assay showed that the tissue levels of pro-inflammatory factors (IL-6, IL-8, and TNF-α) were significantly attenuated by Fetuin-A treatment, by contrast, the anti-inflammatory factor IL-10 was dramatically upregulated after Fetuin-A supplementation (Fig. 4G).

The normal BBB prevents inflammatory cells in blood from invading into the damaged brain tissue, and neutrophil accumulation is another feature of the inflammatory response after the early stage of craniocerebral injury [40]. As the immunostaining showed that the myeloperoxidase (MPO)-positive neutrophils were increased at 6 h following CCI, whereas Fetuin-A treatment reduced the number of MPO-positive neutrophils (Fig. 4H, I).

### Fetuin-A fails to inhibit apoptosis in vitro

**Fig. 5:**
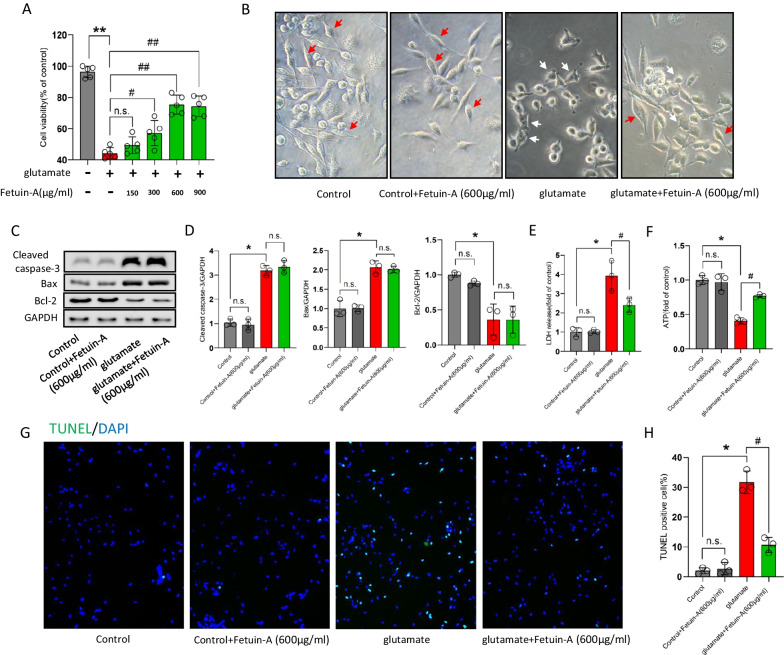
Fetuin-A failure to inhibit apoptosis in vitro. The microglial cells were treated with 100 μm glutamate (Glu) for 24 h to induce cellular injury. Meanwhile, Fetuin-A was treated into medium at indicated concentrations. **A** Cell viability was measured by Cell Counting Kit-8 assay (*n* = 5). **B** Representative photomicrographs of microglial cells in different groups (red arrows represent microglia in normal state and white arrows represent microglia in the activated state). Scale bar is 20 μm. **C**, **D** Expression of cleaved caspase-3, Bax, and Bcl-2 in microglial cells under various treatment conditions. GAPDH was used as the loading control. And bar graphs show the results of analysis (by band density analysis) of these proteins (*n* = 3). **E** LDH release assay (*n* = 3). **F** ATP production was measured by CellTiter-Glo ATP-based luminescence assays (*n* = 3). **G**, **H** Microglial death measured by TUNEL staining. Scale bar is 50 μm (*n* = 7). Data are presented as the means ± SD; *P < 0.05 vs. control, **P < 0.01 vs. control, ^#^*P* < 0.05 vs. Glu group, ^##^*P* < 0.01 vs. Glu group and n.s.: no significant difference

To further explore the molecular and cellular mechanisms of Fetuin-A on inflammatory response following craniocerebral injury, we used the mouse primary microglia to construct a classical in vitro injury model [41]. We first verified that whether Fetuin-A could regulate microglial cells viability. Cell Counting Kit-8 analysis showed that Fetuin-A could affect microglial cells viability in a dose-dependent manner, and treatment with 600 μg/mL of Fetuin-A has an optimal protection to avoid microglial cells death (Fig. 5A). Moreover, Fetuin-A treatment attenuated glutamate-induced inflammatory amoeboid morphology, which suggested that Fetuin-A could inhibit microglial activation in vitro (Fig. 5B). Previous studies have revealed that apoptotic microglia were important for inflammation response [42, 43]. We next examined the level of key apoptosis-related protein by western blot analyses, and the results shown that glutamate treatment increased the apoptotic factors (cleaved caspase-3 and Bax) and decreased the anti-apoptotic factor (Bcl-2). However, the level of the apoptosis-related proteins did not change under Fetuin-A supplementation (Fig. 5C, D). Therefore, Fetuin-A-promoted microglial cells viability was independent of inhibition of apoptosis.

At the same time, we found that glutamate treatment significantly increased the LDH release and decreased the levels of ATP in microglial cells. Interestingly, treating with Fetuin-A reversed the results (Fig. 5E, F). At the same time, TUNEL staining results revealed that Fetuin-A supplementation reduce the numbers of TUNEL-positive cells following glutamate treatment (Fig. 5G, H). In view of those experimental results, we hypothesized that Fetuin-A protects microglial cells after injury by inhibiting necroptosis.

### Fetuin-A produces its anti-inflammatory effect by inhibiting necroptosis in glutamate-treated primary microglia

**Fig. 6:**
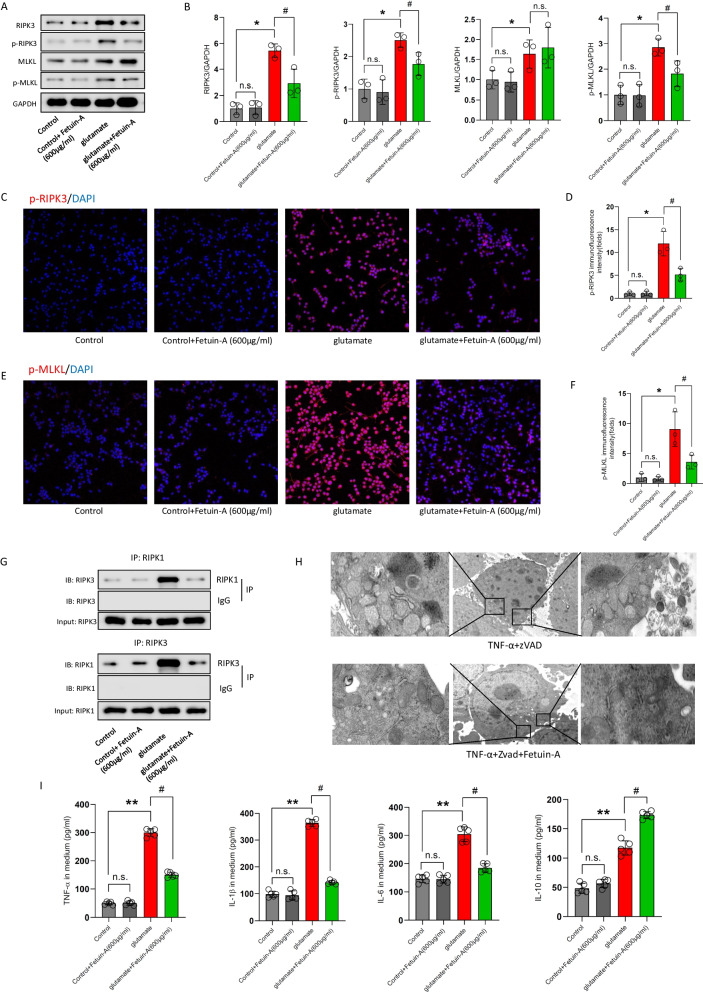
The anti-inflammatory effect of Fetuin-A is by inhibiting necroptosis in glutamate-exposed microglial cells. The microglial cells were treated with 100 μm glutamate (Glu) for 24 h to induce cellular injury. Meanwhile, Fetuin-A was treated into medium at indicated concentrations. **A**, **B** Expression of RIPK3, MLKL, p-RIPK3 and p-MLKL in microglial cells under various treatment conditions. GAPDH was used as the loading control. And bar graphs show the results of analysis (by band density analysis) of these proteins (*n* = 3). **C**–**F** Immunofluorescence assessment of p-RIPK3 and p-MLKL expression. Scale bar is 50 μm. The relative immunofluorescence intensity of Fetuin-A was detected with Image-J software (*n* = 3). **G** RIPK1 was immunoprecipitated with its antibody and resulted in co-immunoprecipitation of RIPK3. Immunoprecipitation of RIPK3 with its antibody caused co-immunoprecipitation of RIPK1 in microglial cells (*n* = 3). **H** Transmission electron microscopy (TEM) images of tissues. Translucent cytoplasm, mitochondrial swelling and destruction of membrane integrity were observed in TNF-α + zVAD-treated microglial cells (scale bar = 5 or 1 μm, *n* = 3). **I** IL-6, IL-1β, TNF-α, and IL-10 secretion was measured using ELISA (*n* = 5). Data are presented as the means ± SD; *P < 0.05 vs. control, **P < 0.01 vs. control, ^#^*P* < 0.05 vs. Glu group, and n.s.: no significant difference

To further verify the mechanisms underlying Fetuin-A-mediated protection in vitro, we first measured the protein expression of necroptosis by western blot analysis. As shown in Fig. 6A, B, the total protein expression of RIPK3 and MLKL was increased in microglial cells at 24 h following glutamate supplementation, moreover, the phosphorylation of RIPK3 and MLKL increased after glutamate treatment. Interestingly, cotreatment with 600 μg/mL of Fetuin-A for 24 h decreased the content of RIPK3, besides, Fetuin-A decreased the phosphorylation of RIPK3 and MLKL. However, Fetuin-A had no significant influence on the expression of MLKL.

And the immunofluorescence staining exhibited that Fetuin-A treatment decreased the immunofluorescence intensity of p-RIPK3 and p-MLKL compare with that in the glutamate group (Fig. 6C–F).

Next, we investigated the interaction between RIPK1 and RIPK3, which promotes the formation of necrosome, in microglial cells by co-immunoprecipitation. While RIPK1 was immunoprecipitated with its antibody, the level of co-immunoprecipitated RIPK3 increased significantly in glutamate-exposed microglial cells, however the Fetuin-A decreased the level of RIPK3 compared with glutamate group. Similarly, immunoprecipitation of RIPK3 with its antibody had much more co-immunoprecipitation of RIPK1 in glutamate-exposed microglial cells, and Fetuin-A abrogates the results (Fig. 6G).

Moreover, transmission electron microscopy (TEM) images exhibited a typical morphology of necroptotic cells, including translucent cytoplasm, mitochondrial swelling and destruction of membrane integrity after treated with TNF-α and apoptosis inhibitor Z-VAD-FMK, which are specific necroptosis inducer [44], and Fetuin-A treatment promoted the morphology (Fig. 6H).

In addition, the ELISA results showed that the increased inflammatory factors following glutamate treatment was reduced by Fetuin- A (Fig. 6I).

### Fetuin-A represses necroptosis by preventing oxidative stress

**Fig. 7:**
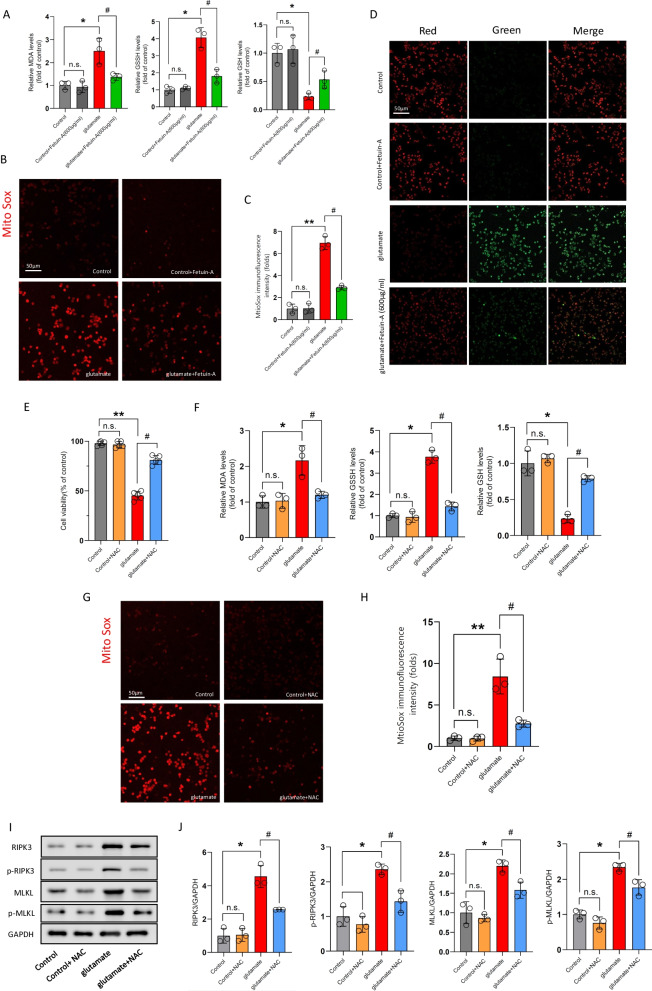
Inhibition of oxidative stress is a key pathway for Fetuin-A to repress necroptosis. The microglial cells were treated with 100 μm glutamate (Glu) for 24 ho to induce cellular injury. **A**, **F** The oxidative stress of microglial cells were analyzed by detecting the levels of MDA, GSSG, and GSH (*n* = 3). **B**, **C**, **G**, **H** Mitochondrial superoxide was detected by immunofluorescence using MitoSox Red staining. Scale bar is 50 μm. The relative immunofluorescence intensity of Fetuin-A was detected with Image-J software (*n* = 3). **D** Mitochondrial membrane potential was detected by the JC-1 fluorescence ratio. Scale bar is 50 μm (*n* = 5). **E** Cell viability was measured by Cell Counting Kit-8 assay (*n* = 5). **I**, **J** Expression of RIPK3, MLKL, p-RIPK3 and p-MLKL in microglial cells under various treatment conditions. GAPDH was used as the loading control. And bar graphs show the results of analysis (by band density analysis) of these proteins (*n* = 3). Data are presented as the means ± SD; *P < 0.05 vs. control, **P < 0.01 vs. control, ^#^*P* < 0.05 vs. Glu group, and n.s.: no significant difference

Microglial cells displayed mitochondrial swelling after TNF-α and Z-VAD-FMK supplementation, which suggested abnormal mitochondrial function. Therefore, we examined oxidative stress indicators in microglial cells following glutamate treatment. As the experimental results showed that the levels of malondialdehyde (MDA), oxidized glutathione (GSSG), which are the markers of oxidative stress, were increased and glutathione (GSH) was decreased in the glutamate-exposed microglial cells, whereas Fetuin-A cotreatment significantly attenuated the above effects (Fig. 7A). Moreover, as shown in MitoSox Red staining, glutamate upregulated the mitochondrial ROS production obviously, which was alleviated by Fetuin-A (Fig. 7B, C). And JC-1 staining results revealed that glutamate induced a dissipation of mitochondrial membrane potential, whereas Fetuin-A ameliorated mitochondrial depolarization (Fig. 7D). These results suggested that Fetuin-A has an antioxidant effect.

Numerous studies in several cell lines such as macrophages, MEFs, and L929 cells have shown that ROS production is necessary for necroptosis [45]. To further investigate the mitochondrial redox-related inter-organelle regulatory mechanism of necroptosis in microglial cells, *N*-acetyl-l-cysteine (NAC, 10 mM, a ROS inhibitor) was used as an antioxidant in glutamate-treated cells.

As shown in Fig. 7E, NAC increased the microglial cells viability following glutamate treatment. Besides, NAC supplementation decreased the level of MDA and GSSG, in contrast, increased the level of GSH compared with the glutamate group (Fig. 7F). In addition, MitoSox Red staining results shown that NAC downregulated mitochondrial superoxide following glutamate treatment (Fig. 7G, H), suggesting that NAC avoided microglial cells death and inhibited the oxidative stress in glutamate-exposed microglial cells. In addition, western blot analysis revealed that the protein expression of RIPK3 and MLKL were decreased in glutamate + NAC group compared with glutamate group (Fig. 7I, J), and NAC treatment decreased the phosphorylation of RIPK3 and MLKL. Those results indicated mitoROS intervention reduced the level of necroptosis in microglial cells after injury.

### The Nrf-2/HO-1 pathway is involved in the antioxidant mechanism of Fetuin-A in vitro

**Fig. 8:**
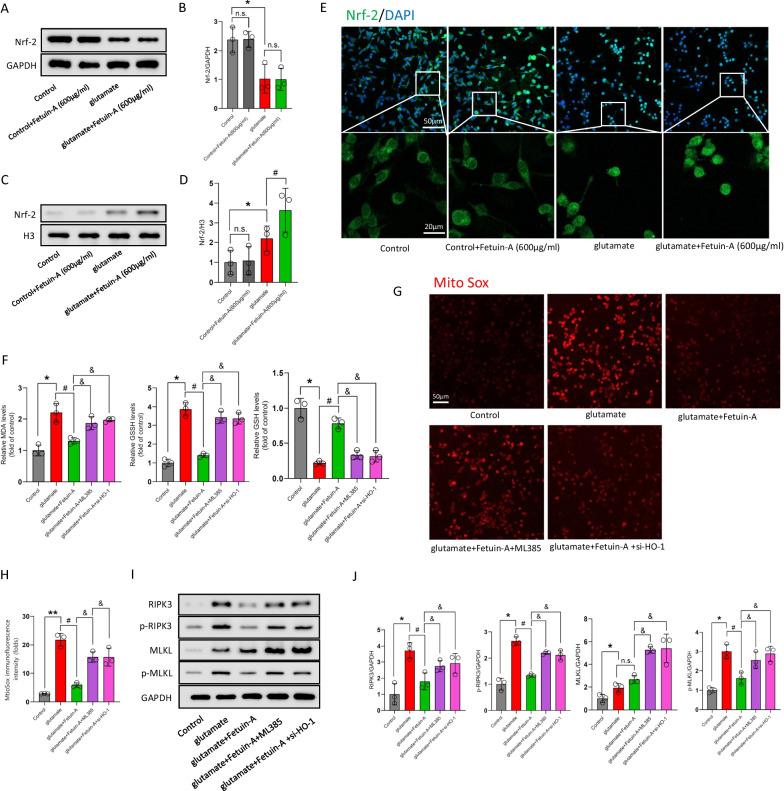
Nrf-2/HO-1 pathway is involved in the protection mechanism of Fetuin-A in glutamate-treated microglial cells. **A**–**D** Western blot analysis of Nrf-2 expression in cytosolic or nuclear. GAPDH and Histone H3 were used as the loading control. And bar graphs show the results of analysis (by band density analysis) of these proteins (*n* = 3). **E** The intracellular localization of Nrf-2 was visualized by confocal microscopy. Scale bar is 20 or 50 μm (*n* = 5). **F** Oxidative stress of microglial cells were analyzed by detecting the levels of MDA, GSSG, and GSH (*n* = 3). **G**, **H** Mitochondrial superoxide was detected by immunofluorescence using MitoSox Red staining. Scale bar is 50 μm. The relative immunofluorescence intensity of Fetuin-A was detected with Image-J software (*n* = 3). **I**, **J** Detection of RIPK3, MLKL, p-RIPK3 and p-MLKL was done by immunoblot analysis (*n* = 3). Data are presented as the means ± SD; *P < 0.05 vs. control, **P < 0.01 vs. control, ^#^*P* < 0.05 vs. Glu group, ^&^*P* < 0.05 vs Glu + Fetuin-A group, and n.s.: no significant difference

Nrf-2 enhances HO-1 transcription, which is involved in an antioxidant pathway, by binding to the antioxidant response element in the nucleus. In view of the pivotal role of Nrf-2/HO-1 in oxidative stress, we next investigated whether nuclear translocation of Nrf-2 and transcription of HO-1 are involved in the protective mechanism of Fetuin-A against oxidative stress. We performed western blot assessments, and found that the Nrf2 expression in cytosol was not affected by Fetuin-A following glutamate treatment, however, Fetuin-A increased the nuclear Nrf2 content compared with glutamate group (Fig. 8A–D). In congruent, the results of the immunofluorescence exhibited similar experimental results (Fig. 8E).

To further explore the regulatory mechanism involved in Fetuin-A, we administered microglial cells with ML385, which is the Nrf-2 inhibitor. We found that ML385 significantly inhibit Nrf-2 expression after 72 h at the concentrations of 8 and 10 μmol/L (Additional file 5: Fig. S5A, B). However, given that ML385 may cause cytotoxicity at high concentration, we chose 8 μmol/L as the final intervention concentration of ML385. Besides, we designed the HO-1 knockdown expression microglial-siHO-1 cells, and the first sequence proved to be the most efficient, therefore we used it in all following experiments (Additional file 5: Fig. S5C, D). We repeated the above experiments about oxidative stress. The results showed that Fetuin-A reduced the levels of MDA, GSSG and increased GSH in glutamate-treated microglial cells, but as expected, ML385 or si-HO-1 blocked the effect of Fetuin-A, and the level of oxidative stress increased significantly after downregulated the Nrf-2 or HO-1 (Fig. 8F). Similarly, Fetuin-A had no obvious effect on mitochondrial superoxide in glutamate-treated microglial cells which were co-treated with ML385 or si-HO-1 (Fig. 8G, H).

Moreover, the reduction effect of Fetuin-A on the total protein expression and phosphorylation ofRIPK3 and MLKL is limited in microglial cells, which administered ML385 or si-HO-1, following glutamate treatment by western blot analysis (Fig. 8I, J). These results indicated that knockdown of Nrf-2/HO-1 pathway could suppressed the protection of Fetuin-A on oxidative stress and necroptosis after glutamate treatment.

### The therapeutic effect of Fetuin-A was related to the inhibition of microglial activation

**Fig. 9:**
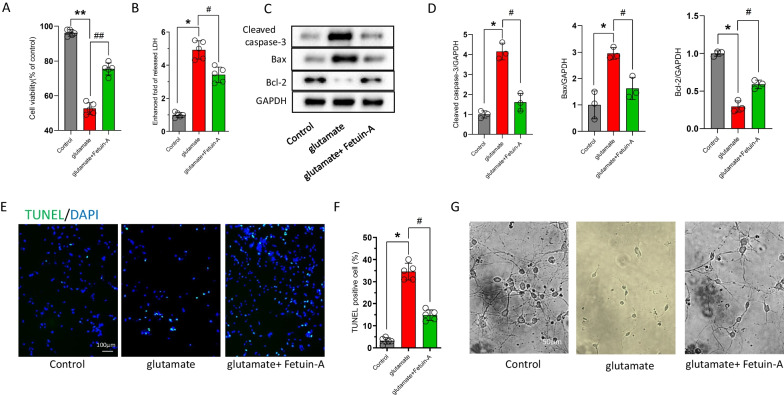
Fetuin-A suppressed neuronal damage in vitro. The primary cortical neurons were cultured in neurobasal medium with 2% B27 and 0.5 mM glutamate. Then, the neurons were treated with the microglia-conditioned medium. **A** Cell viability was measured by Cell Counting Kit-8 assay (*n* = 5). **B** LDH release assay (*n* = 5). **C**, **D** Western blot analysis of the apoptosis-related proteins expression. GAPDH was used as the loading control. And bar graphs show the results of analysis (by band density analysis) of these proteins (*n* = 3). **E**, **F** Cell death measured by TUNEL staining of primary neurons. Scale bar is 100 μm (*n* = 5). **G** Representative images of neurons growth 1 week after different treatments. Scale bar is 50 μm (*n* = 5). Data are presented as the means ± SD; *P < 0.05 vs. control, **P < 0.01 vs. control, ^#^*P* < 0.05 vs. Glu group

Given the involvement of Fetuin-A in the inhibition of the inflammatory response in glutamate-treated microglial cells, we added microglia-conditioned medium from the above groups to primary cortical neurons. The results of cell viability (Fig. 9A) and LDH release (Fig. 9B) showed that Fetuin-A had an obvious protective effect on neurons. Next, we examined the levels of apoptosis-related proteins by western blot, and the results demonstrated that the apoptotic factors (cleaved caspase-3 and Bax) were increased and the anti-apoptotic factor (Bcl-2) was decreased in the glutamate-treated group; whereas, the levels of the apoptosis-related proteins were reversed in the glutamate + Fetuin-A group (Fig. 9C, D). Moreover, neuronal apoptosis was detected by TUNEL staining (Fig. 9E, F). In addition, we examined the morphology of neurons after 7 days. Neurons displayed axonal shortening and aggregation in the glutamate-treated group, and Fetuin-A ameliorated these changes (Fig. 9G).

**Fig. 10:**
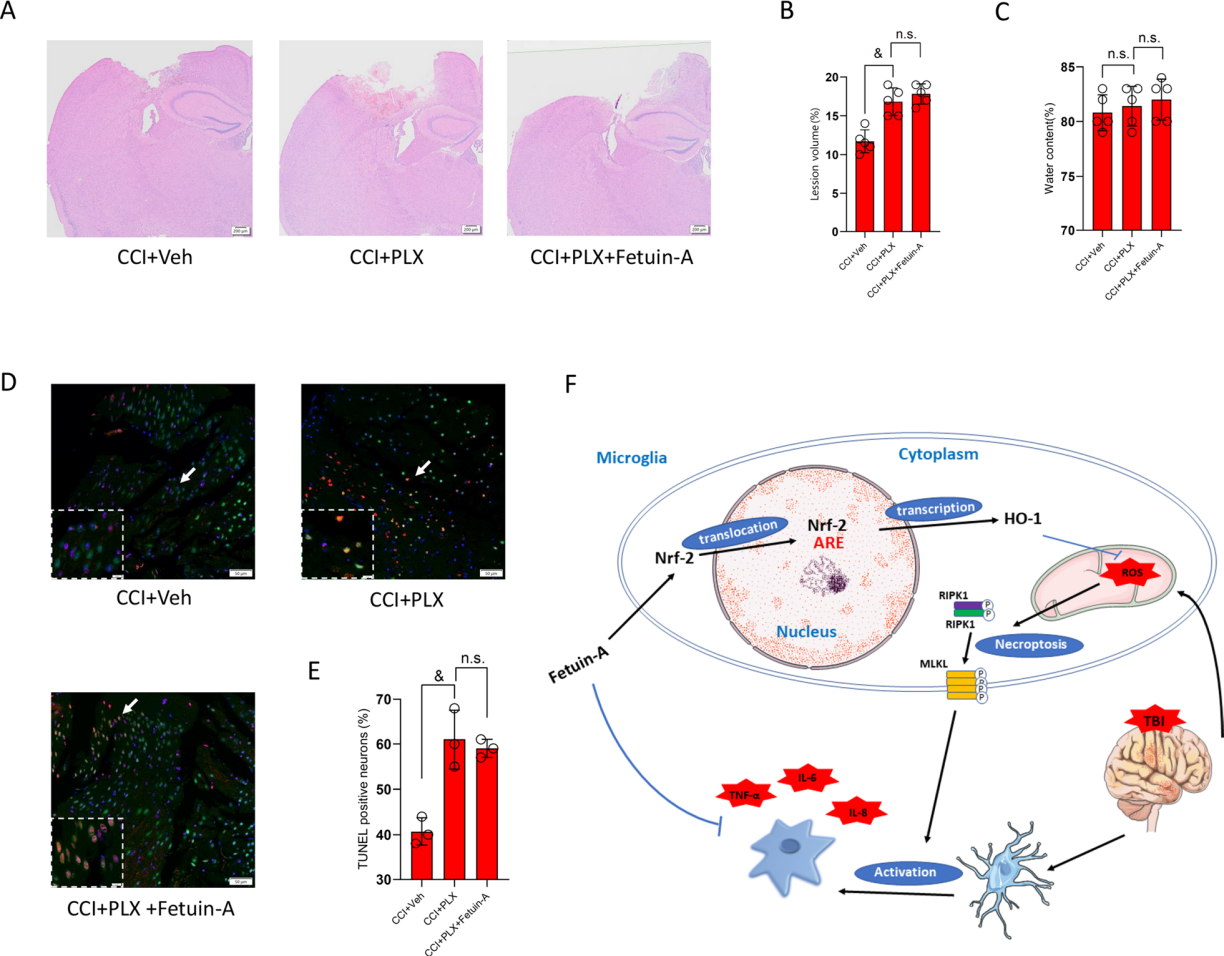
PLX5622-mediated microglia depletion attenuates the effect of Fetuin-A in TBI mice. **A** H&E staining of hemispheres sections (*n* = 3). **B**, **C** Lesion volume (*n* = 5) and water content% (*n* = 5) were analyzed by statistical. **D**, **E** Neuron death measured by TUNEL staining. Scale bar is 50 μm (*n* = 3). **F** A schematic diagram showing the neuroprotective and anti-inflammation effects of Fetuin-A. Data are presented as the means ± SD; ^&^*P* < 0.05 vs. CCI + Veh group, and n.s.: no significant difference

Next, we investigated whether the therapeutic effect of Fetuin-A was dependent on the presence of microglia following CCI in vivo. As described previously, PLX5622, which is a specific CSF1R inhibitor, robustly eliminates microglial cells brain-wide with low side effects on other CNS-resident cells [46, 47]. Mice were started on dietary administration of PLX5622 2 weeks before CCI, which caused a 90% depletion of Iba-1-positive cells compared with mice administered with AIN-76A standard chow (Additional file 6: Fig. S6A–D). We next subjected the mice to CCI injury with or without Fetuin-A treatment. As shown by Fig. 10A, B, the cortical lesion volume and edema area in the CCI + PLX group were significantly larger than those in the CCI + Veh group, however, Fetuin-A-treated group had no significant improvement compared with the CCI + PLX group. And, there was no significant difference in brain water content between the CCI + PLX group and CCI + PLX + Fetuin-A group (Fig. 10C). Consistently, the results of TUNEL assay exhibited that the anti-apoptotic effect of Fetuin-A was lost when microglial cells were depleted (Fig. 10D–E).

Together, these results suggested that the neuroprotective effect of Fetuin-A might be related to the involvement of microglia activation.

## Discussion

Surgery is the first-line treatment for severe TBI in clinical practice, but postoperative patients still face a series of symptoms such as disturbance of consciousness, decreased motor and sensory function, and neuromodulatory disorders, which are related to neuronal death [34, 36]. However, few effective therapeutic treatments have been developed to inhibit secondary injury after TBI, and improving the prognosis of patients is an urgently clinical problem. In the current study, we found that Fetuin-A has a neuroprotective effect in TBI. Mechanistically, we demonstrated that Fetuin-A can exert anti-inflammatory effects by inhibiting microglial activation, reducing formation of necrosome complexes, and suppressing oxidative stress. Furthermore, we revealed that Fetuin-A increased the nuclear localization of Nrf-2 and HO-1 expression in glutamate-treated microglial cells, whereas treatment with ML385, an inhibitor of Nrf-2, or HO-1 knockdown inhibited the effects of Fetuin-A on oxidative stress and necroptosis. Finally, Fetuin-A supplementation reduce the release of cytotoxic substances from damaged microglia, thereby alleviating neuronal death. In addition, the elimination of microglia by PLX5622 further confirms that microglia might be the target cells for Fetuin-A treatment and the efficacy was associated with increased microglial activation (Fig. 10F).

Previous studies have revealed that Fetuin-A attenuates the inflammatory response and protects against injury in cerebral ischemia, intestinal ischemia/reperfusion and hereditary angioedema [38, 48, 49]. And according to our GO analysis, Fetuin-A is predicted to be involved in the biological processes of “acute phase response” and “inflammation”. Meanwhile, according to previous research findings, the levels of Fetuin-A markedly increased at 24 h, and peaked at 48 h in mouse ischemic brain tissue compared with the wild-types [38]. However, the data on Fetuin-A are rarely mentioned in the study of craniocerebral injury. Our experimental results shown that the level of Fetuin-A peaked at 6 h following CCI, which was different from the peak at 48 h in cerebral ischemia. And, we speculated that the temporal peak of Fetuin-A may vary because of differences in the mouse models used. Therefore, it is necessary to determine whether Fetuin-A has anti-inflammatory effects following TBI and the associated mechanism.

Microglia are thought to remain in a characteristic quiescent state with high motility to maintain the stability of the CNS microenvironment under physiological conditions. However, TBI triggers rapid activation of microglia, which tend to displayed morphological features such as reduced process length, branch endpoint and swollen soma, thus releasing large amounts of inflammatory mediators that act on other cells and induce a cascading inflammatory response [50–52]. Therefore, a better understanding of the microglial activation mechanism may provide a potential therapeutic strategy. Our study showed that Fetuin-A treatment inhibited microglial activation. Consistently, Fetuin-A administration attenuated release of the pro-inflammatory and prevented the invasion of neutrophils into the peri-contusional area. We previously reported that necroptosis is involved in inflammatory response and induces neural damage, and p-RIPK3 and p-MLKL constitute the core signaling pathway involved in the induction of necroptosis [7]. In the present study, we found that Fetuin-A decreased the content of RIPK3 and the phosphorylation of RIPK3 and MLKL, and inhibited the aggregation of RIPK1 and RIPK3 into necrosomes. However, Fetuin-A had no significant influence on the expression of MLKL. Recently, more and more researches have paid attention to the mechanisms of necroptosis, and increasing evidence has demonstrated that ROS accumulation induces necroptosis, and inhibition of ROS suppresses RIP-mediated necroptosis [20, 53]. In this study, we found that Fetuin-A treatment significantly attenuated intracellular ROS accumulation and the ROS scavenger (NAC) decreased p-RIPK3 and p-MLKL protein expression after injury. These results show that Fetuin-A has an antioxidant effect and that ROS accumulation induces necroptosis in microglia under pathological conditions. Moreover, increasing evidenced have shown that Nrf-2 exerted protective effects against oxidative stress, inflammation, and cell death in nervous system disease [54, 55]. Activated Nrf-2 translocates to the nucleus and initiates transcription of various genes, specifically HO-1 [21]. Congruently, our study showed that Fetuin-A further increased Nrf-2 content in the nucleus, therefore enhanced transcription of HO-1 after injury. In addition, we observed the ROS accumulation and necroptosis were obviously increased following Nrf-2 or HO-1 down-regulation. These findings showed that Fetuin-A protects against necroptosis and oxidative stress by activating the Nrf-2/HO-1 system. To determine whether the protective effect of Fetuin-A depended on the presence of microglia after CCI, we eliminate microglial cells brain-wide via PLX5622. And we found that the protective effect of Fetuin-A on the central nervous system is limited when microglia were eliminated. These results demonstrated that the anti-inflammatory effects of Fetuin-A mainly target microglia and protect neurons by inhibiting microglia activation after craniocerebral injury.

However, there are several limitations to this study. First, our microglial injury model was induced by glutamate. Although this is a widely used model, the physiological and pathological processes may differ from those of traumatic injury. Second, we demonstrated the anti-inflammatory and anti-oxidative stress effects of Fetuin-A following CCI; however, the other potential mechanisms of Fetuin-A are also worth investigating in future studies. Third, it would be worthwhile to explore the molecular mechanism of Fetuin-A in regulation of the Nrf-2/HO-1 pathway. In addition, there are many problems associated with treating neurological diseases by intravenous administration because of the unique anatomical and physiological characteristics of the BBB, and although we demonstrated that the state of injury of BBB dysfunction persists into the subacute phase after traumatic injury, intrathecal administration may be a more effective clinical treatment.

## Conclusions

In summary, we demonstrate that Fetuin-A supplementation protects against TBI-induced cellular damage and functional disturbances via its anti-inflammatory and anti-oxidative stress effects. Furthermore, activation of the Nrf-2/HO-1 pathway is an important molecular mechanism by which Fetuin-A prevents CNS injury. Therefore, our findings suggest Fetuin-A as a potential therapeutic agent for intervention in TBI. In addition, this study emphasizes the necessity of targeting inflammation and exploring drugs that can alleviate the abnormal inflammatory response following TBI. Identified drugs can be used for comprehensive clinical treatment of patients after TBI and other acute CNS injuries.

## Supplementary Information

**Additional file 1: Fig. S1.** Experimental design. A. Experiment was designed to identify global differences in protein expression between CCI and sham mouse groups. B. Experiment was designed to determine the endogenous Fetuin-A at each time point. C. Experiment was designed to detect the content of Fetuin-A after tail-vein Injection. D. Experiment was designed to detect whether intravenous administration of Fetuin-A could cross the intact BBB. E. Experiment was designed to test whether intravenous administration of Fetuin-A has biological toxicity. F. Experiment was designed to determine the extent and duration of BBB damage after CCI. G. Experiment was designed to detect the role of Fetuin-A in CCI model and explore the underlying mechanism of Fetuin-A. H. Experiment was designed to detect interference efficiency of AD-shFetuin-A and examine if the injury is worse by blocking Fetuin-A after CCI. I. Experiment was designed to detect the efficiency of microglia depletion and whether the therapeutic effect of Fetuin-A was dependent on the presence of microglia following CCI in vivo.

**Additional file 2: Fig. S2.** A, B. Western blot analysis of ZO-1 and Occludin expression from peri-contusional area. GAPDH was used as the loading control. And bar graphs show the results of analysis (by band density analysis) of these proteins (*n* = 3). C. The co-localization of ZO-1 or Occludin with CD31 by immunofluorescence assay with representative imaging. Scale bar = 10 μm (*n* = 3). Data are presented as the means ± SD; *P < 0.05 vs. sham, and n.s.: no significant difference.

**Additional file 3: Fig. S3.** A-B. Western blot analysis of Fetuin-A expression. GAPDH was used as the loading control. And bar graphs show the results of analysis (by band density analysis) of these proteins (*n* = 3). C. H&E staining of hemispheres sections (*n* = 3). D. water content% (*n* = 3) were analyzed by statistical. E–F. cell death measured by TUNEL staining. Scale bar is 100 μm (*n* = 3). Data are presented as the means ± SD; n.s.: no significant difference.

**Additional file 4: Fig. S4.** A, B. Western blot analysis of Fetuin-A expression after transfected with shFetuin-A. GAPDH was used as the loading control. And bar graphs show the results of analysis (by band density analysis) of these proteins (*n* = 3). C. H&E staining of hemispheres sections (*n* = 3). D, E. Lesion volume (*n* = 5) and water content% (*n* = 5) were analyzed by statistical. F, G. Neuron death measured by TUNEL staining. Scale bar is 50 μm (*n* = 5). Data are presented as the means ± SD; *P < 0.05 vs. sham group, **P < 0.01 vs. sham group, ^#^*P* < 0.05 vs. CCI + shCtrl group, and n.s.: no significant difference.

**Additional file 5: Fig. S5.** A–D. Western blot analysis of Nrf-2 or HO-1 expression after transfected with ML385 or siHO-1, GAPDH were used as the loading control. And bar graphs show the results of analysis (by band density analysis) of these proteins (*n* = 3). Data are presented as the means ± SD; *P < 0.05 vs. control, ^#^*P* < 0.05 vs. Glu + siCtrl group, and n.s.: no significant difference

**Additional file 6: Fig. S6.** PLX5622, which was specific CSF1R inhibitor, was able to achieve robust brain-wide microglia elimination. We fed PLX5622 to mice and tested its elimination efficiency. A, B. Immunofluorescence for Iba-1 in the hemisphere and statistical analysis of Iba-1. Scale bar is 100 μm (*n* = 3). Data are presented as the means ± SD; ***P < 0.001 vs. sham + Veh group, ^###^*P* < 0.001 vs. CCI + Veh group, and n.s.: no significant difference

## Data Availability

The data used to support the findings of this study are available from the corresponding author upon request.

## Abbreviations

TBI: Traumatic brain injury
CCI: Controlled cortical impact
TEM: Transmission electron microscopy
Nrf-2: Nuclear factor erythroid 2-related factor 2
HO-1: Heme oxygenase-1
CNS: Central nervous system
TNF-α: Tumor necrosis factor alpha
RIPK1: Receptor-interacting protein 1
RIPK3: Receptor-interacting protein 3
MLKL: Mixed lineage kinase domain-like pseudokinase
LC–MS: Label-free liquid chromatography–mass spectrometry
ROS: Reactive oxygen species
MDA: Malondialdehyde
GSSG: Oxidized glutathione
GSH: Glutathione
NAC: *N*-Acetyl-l-cysteine
Glu: Glutamate
H&E: Hematoxylin and eosin
TUNEL: Terminal deoxynucleotidyl transferase mediated dUTP nick end labeling
ELISA: Enzyme-linked immunosorbent assay
LDH: Lactate dehydrogenase
MPO: Myeloperoxidase
CSF1R: Colony-stimulating factor 1 receptor
