# Supplementary figures and images for: Fetuin-A alleviates neuroinflammation against traumatic brain injury-induced microglial necroptosis by regulating Nrf-2/HO-1 pathway
Source: J Neuroinflammation. 2022 Nov 4;19:269. doi: 10.1186/s12974-022-02633-5 (PMC9636801; doi:10.1186/s12974-022-02633-5)

Supplementary Figure 1

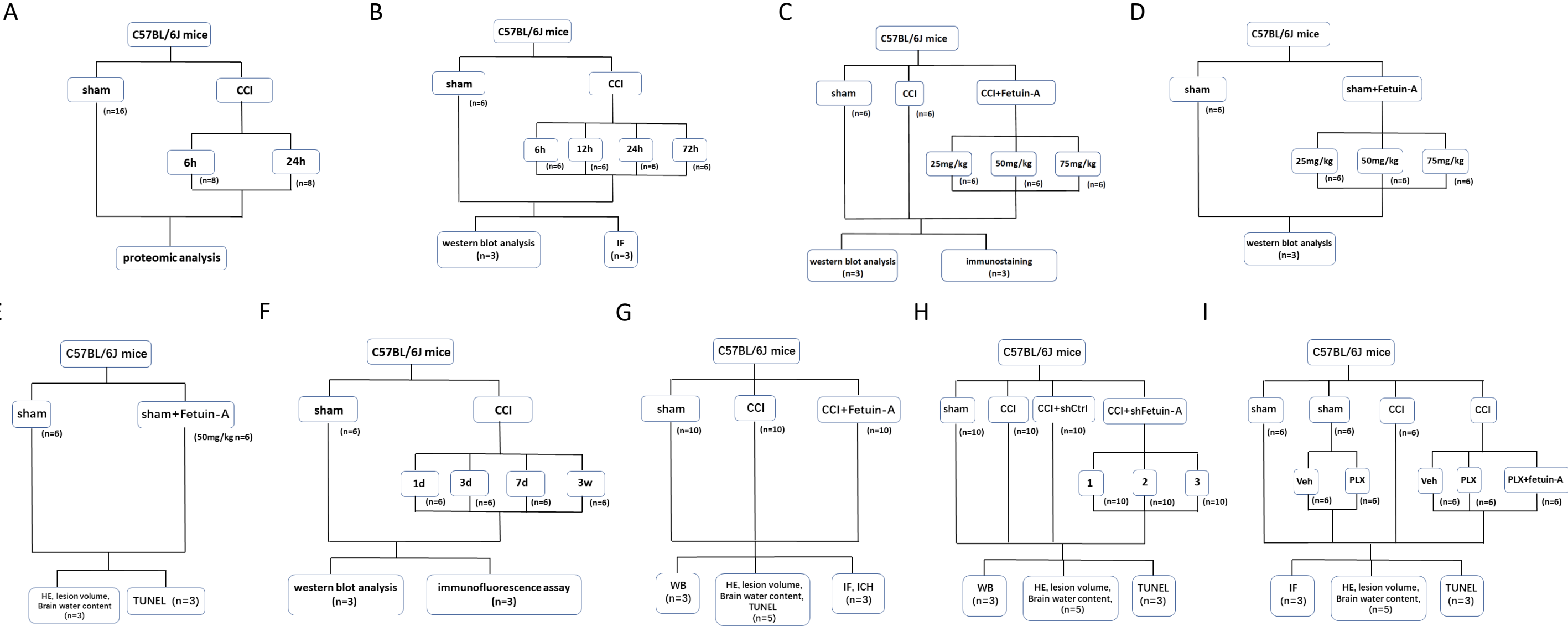

Supplement: Supplementary file 1 — Additional file 1: Fig. S1. Experimental design. A. Experiment was designed to identify global differences in protein expression between CCI and sham mouse groups. B. Experiment was designed to determine the endogenous Fetuin-A at each time point. C. Experiment was designed to detect the content of Fetuin-A after tail-vein Injection. D. Experiment was designed to detect whether intravenous administration of Fetuin-A could cross the intact BBB. E. Experiment was designed to test whether intravenous administration of Fetuin-A has biological toxicity. F. Experiment was designed to determine the extent and duration of BBB damage after CCI. G. Experiment was designed to detect the role of Fetuin-A in CCI model and explore the underlying mechanism of Fetuin-A. H. Experiment was designed to detect interference efficiency of AD-shFetuin-A and examine if the injury is worse by blocking Fetuin-A after CCI. I. Experiment was designed to detect the efficiency of microglia depletion and whether the therapeutic effect of Fetuin-A was dependent on the presence of microglia following CCI in vivo. [file 12974_2022_2633_MOESM1_ESM.pdf]

A

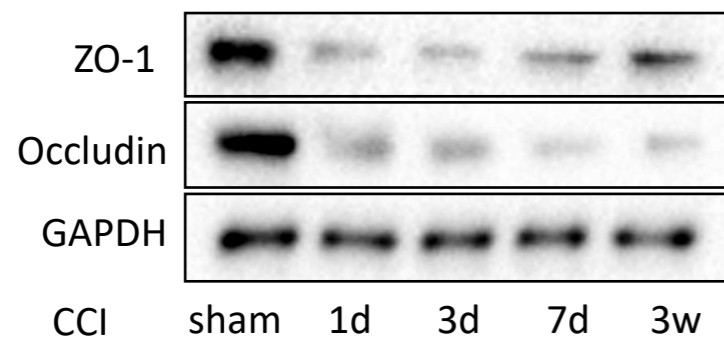

B

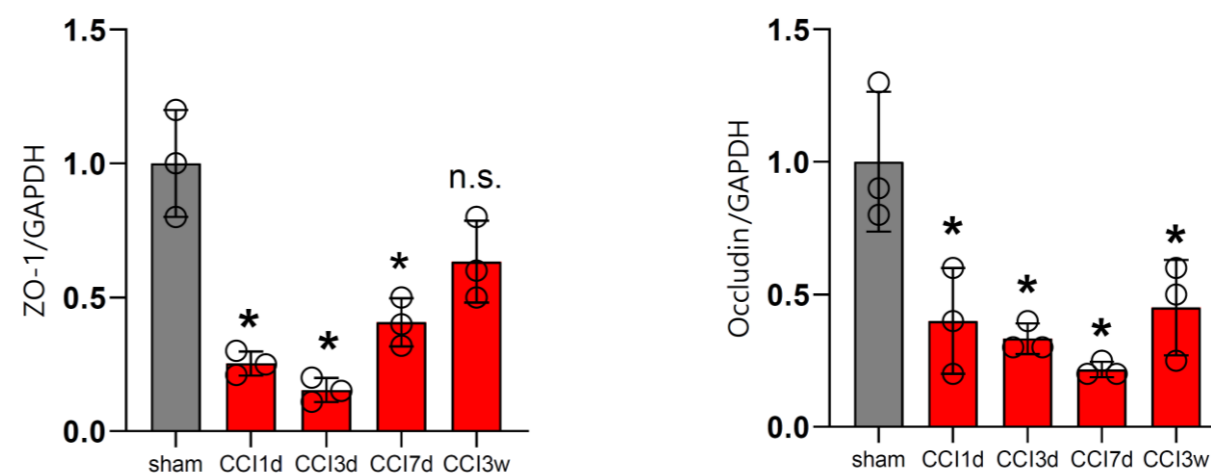

C

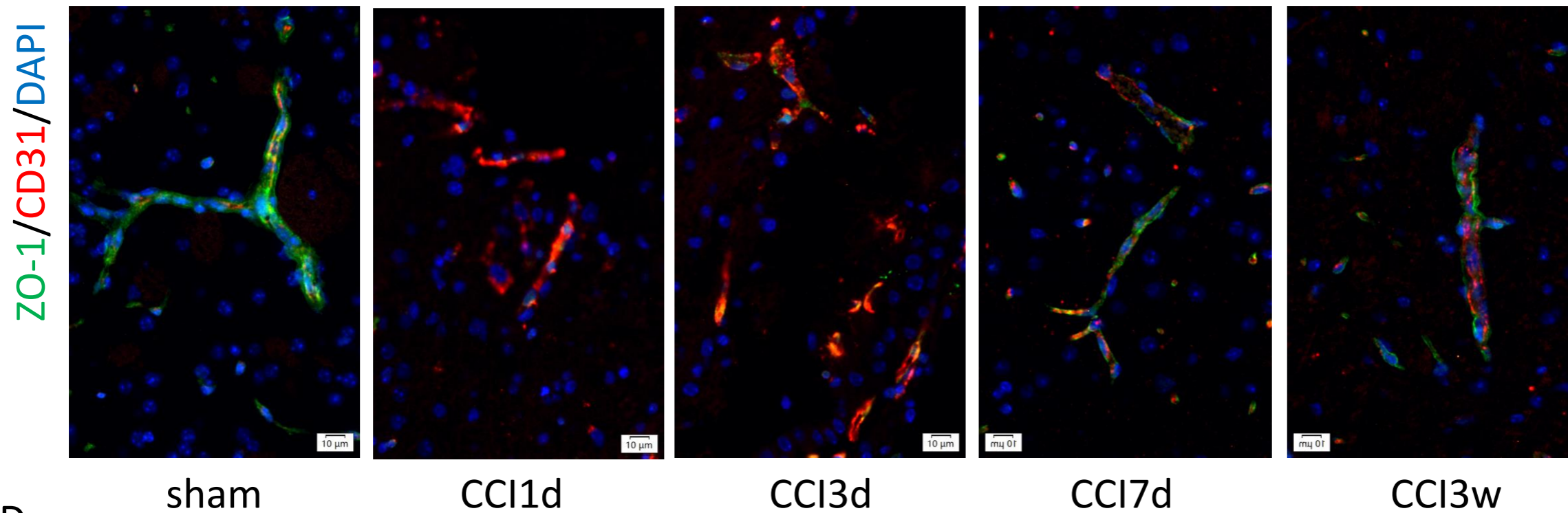

D

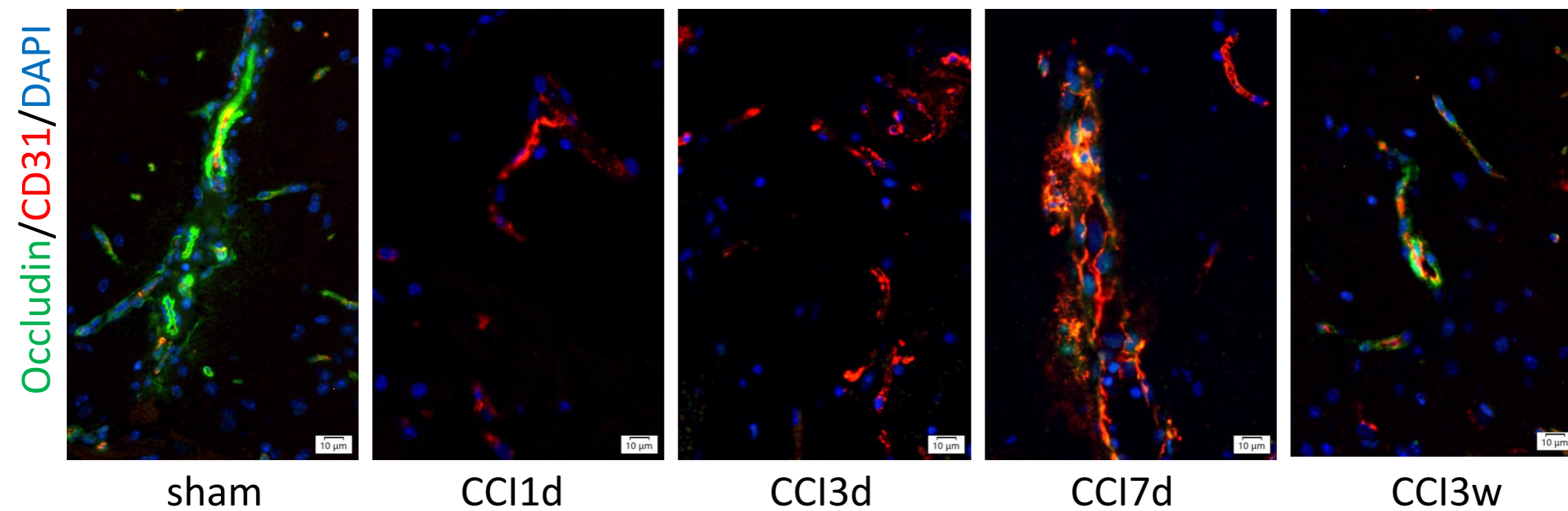

Supplement: Supplementary file 2 — Additional file 2: Fig. S2. A, B. Western blot analysis of ZO-1 and Occludin expression from peri-contusional area. GAPDH was used as the loading control. And bar graphs show the results of analysis (by band density analysis) of these proteins (n = 3). C. The co-localization of ZO-1 or Occludin with CD31 by immunofluorescence assay with representative imaging. Scale bar = 10 μm (n = 3). Data are presented as the means ± SD; *P < 0.05 vs. sham, and n.s.: no significant difference. [file 12974_2022_2633_MOESM2_ESM.pdf]

A

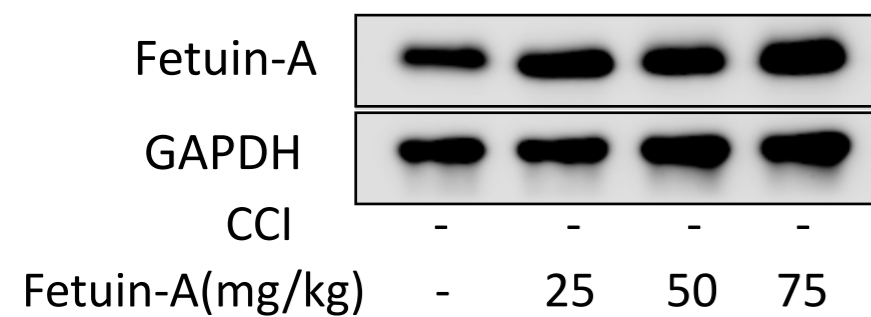

B

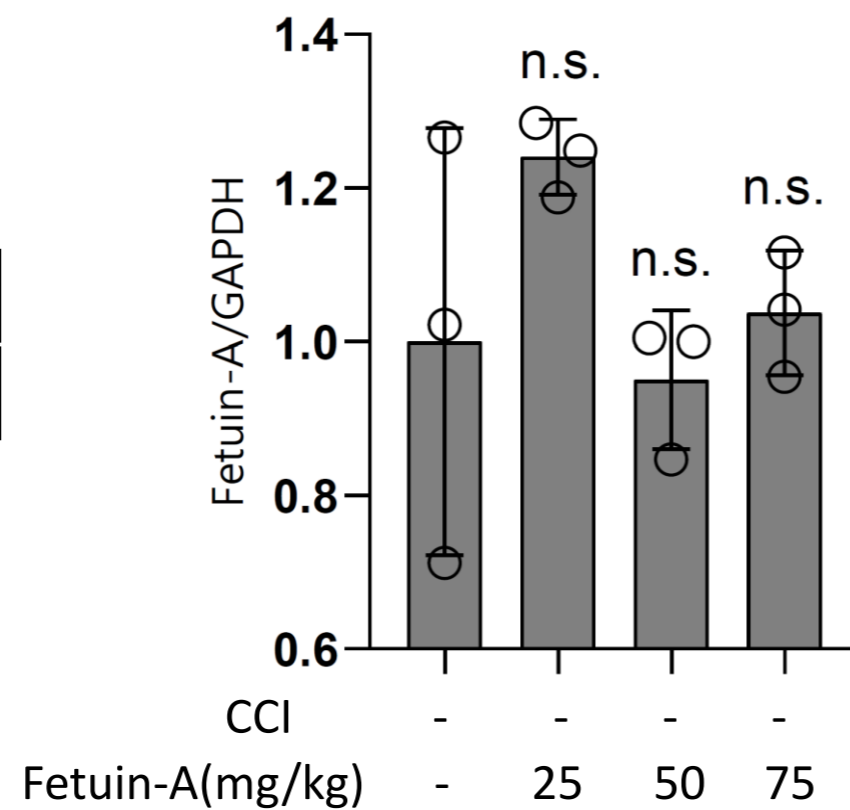

C

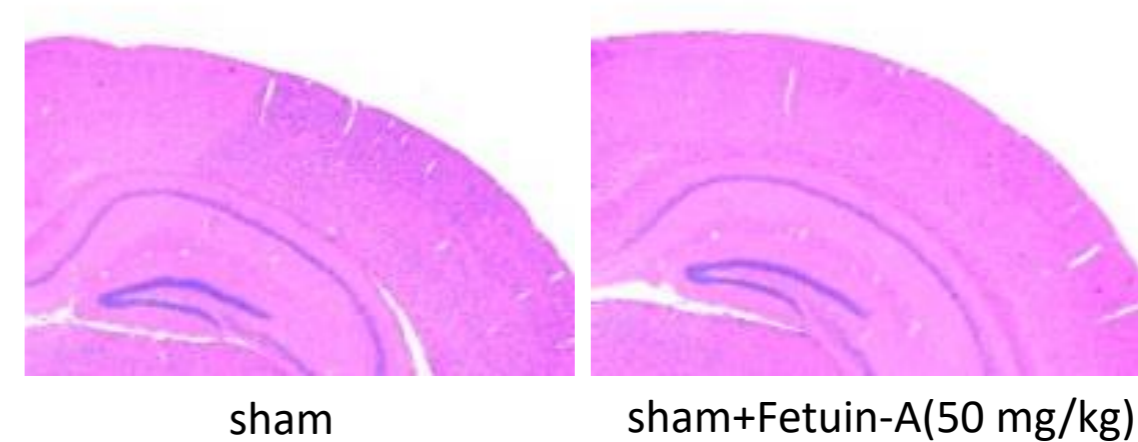

D

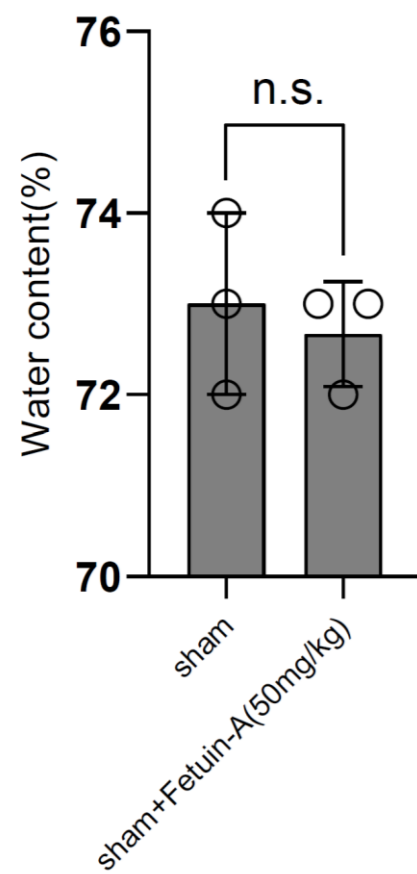

E

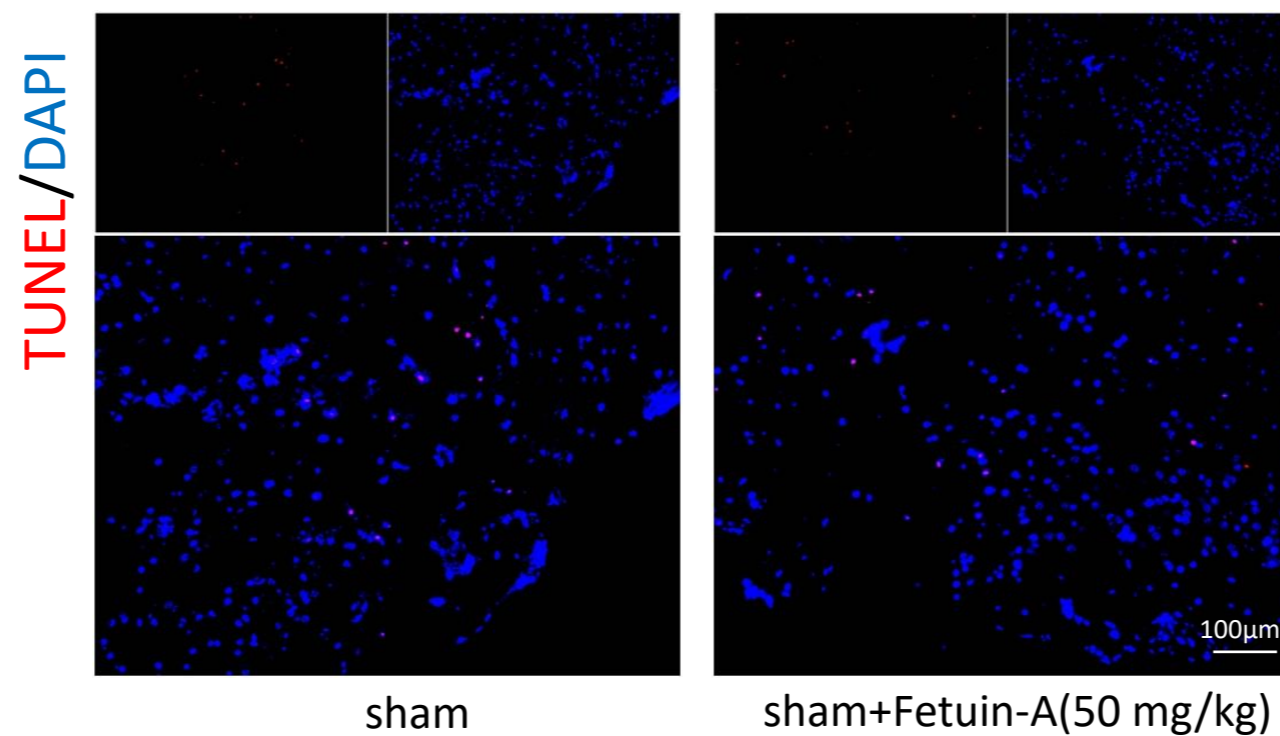

F

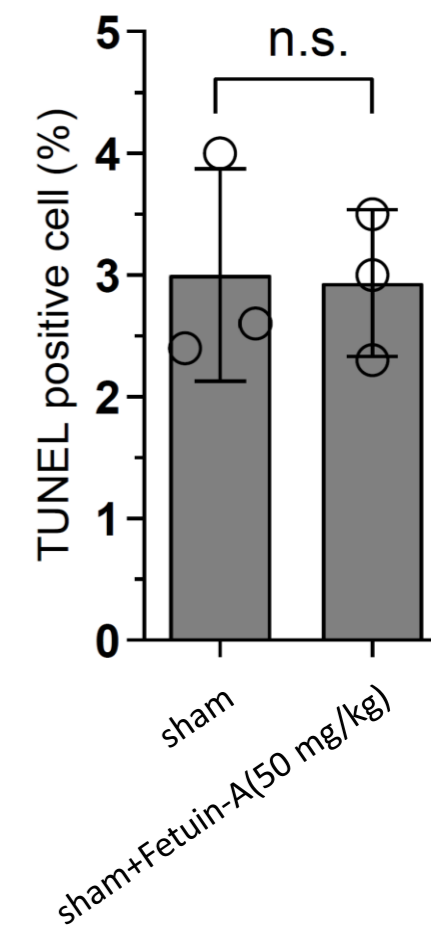

Supplement: Supplementary file 3 — Additional file 3: Fig. S3. A-B. Western blot analysis of Fetuin-A expression. GAPDH was used as the loading control. And bar graphs show the results of analysis (by band density analysis) of these proteins (n = 3). C. H&E staining of hemispheres sections (n = 3). D. water content% (n = 3) were analyzed by statistical. E–F. cell death measured by TUNEL staining. Scale bar is 100 μm (n = 3). Data are presented as the means ± SD; n.s.: no significant difference. [file 12974_2022_2633_MOESM3_ESM.pdf]

A

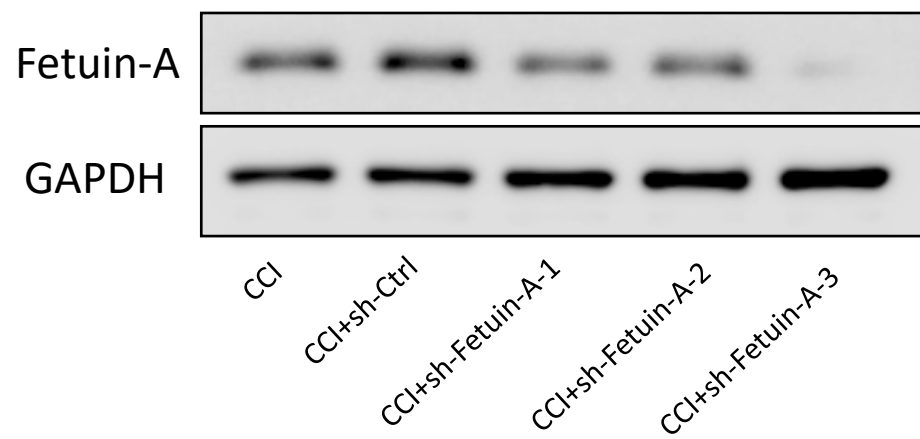

B

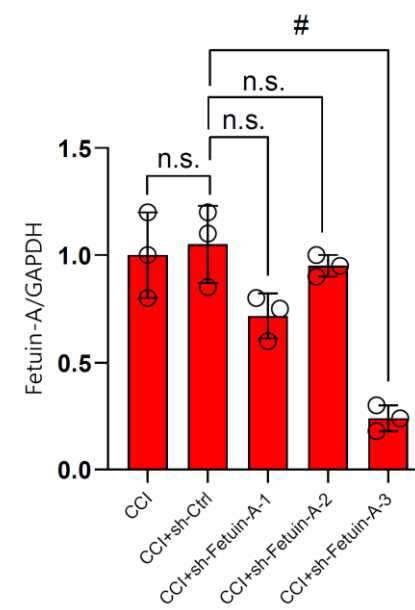

C

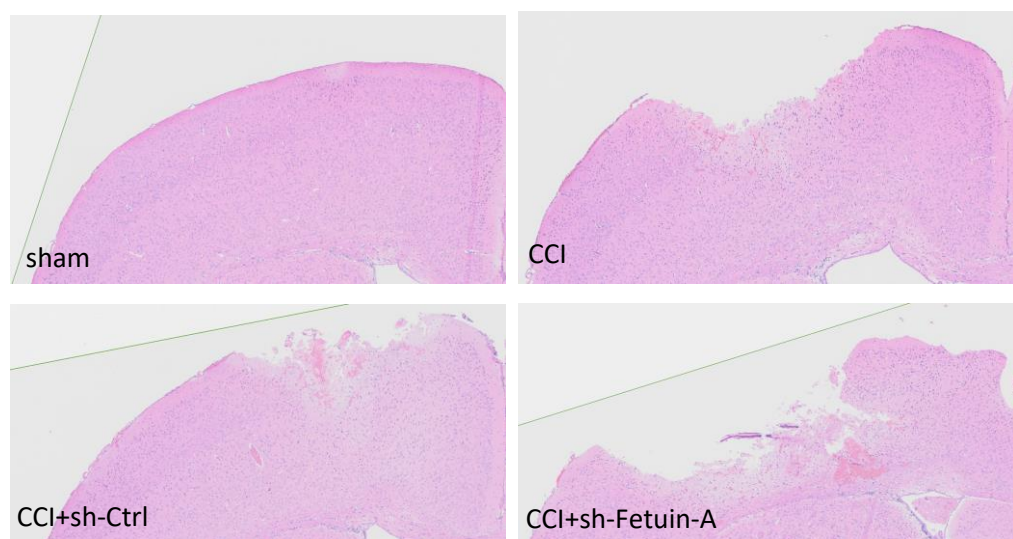

D

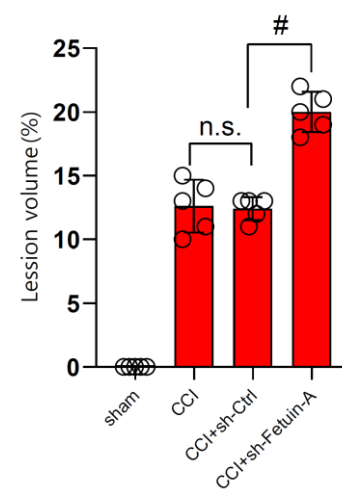

E

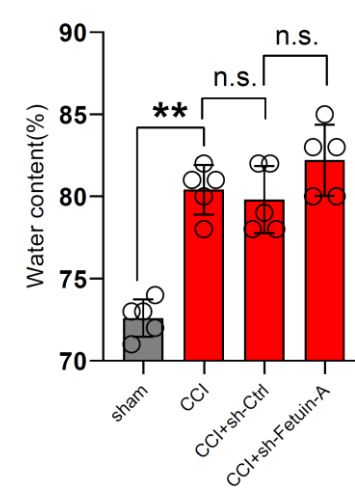

G

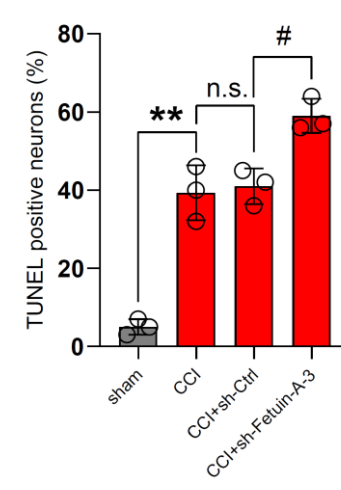

F

TUNEL/NeuN/DAPI

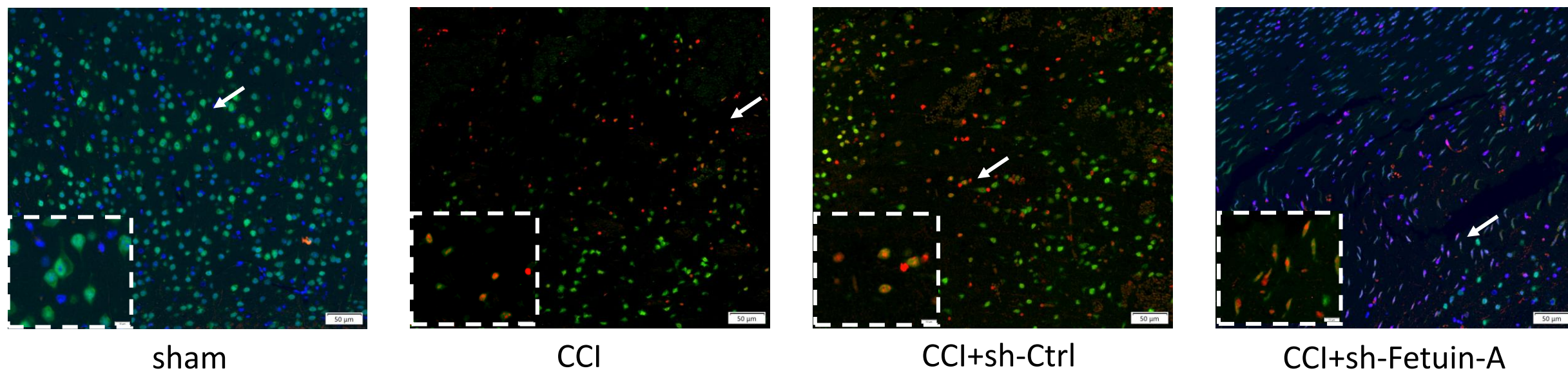

Supplement: Supplementary file 4 — Additional file 4: Fig. S4. A, B. Western blot analysis of Fetuin-A expression after transfected with shFetuin-A. GAPDH was used as the loading control. And bar graphs show the results of analysis (by band density analysis) of these proteins (n = 3). C. H&E staining of hemispheres sections (n = 3). D, E. Lesion volume (n = 5) and water content% (n = 5) were analyzed by statistical. F, G. Neuron death measured by TUNEL staining. Scale bar is 50 μm (n = 5). Data are presented as the means ± SD; *P < 0.05 vs. sham group, **P < 0.01 vs. sham group, #P < 0.05 vs. CCI + shCtrl group, and n.s.: no significant difference. [file 12974_2022_2633_MOESM4_ESM.pdf]

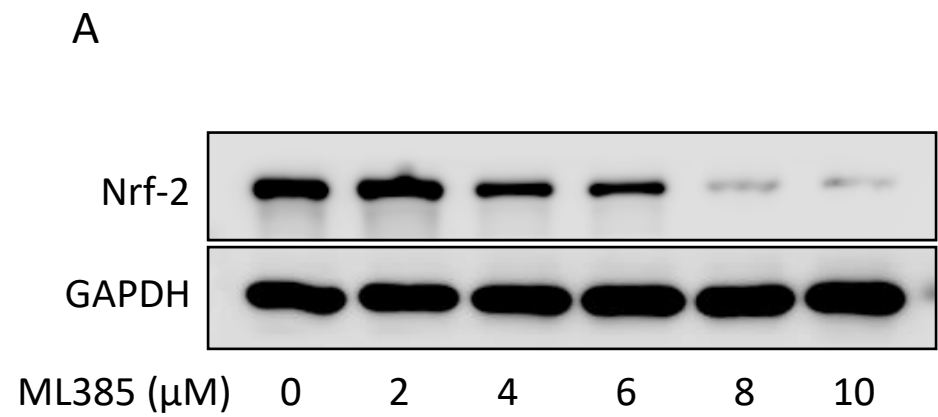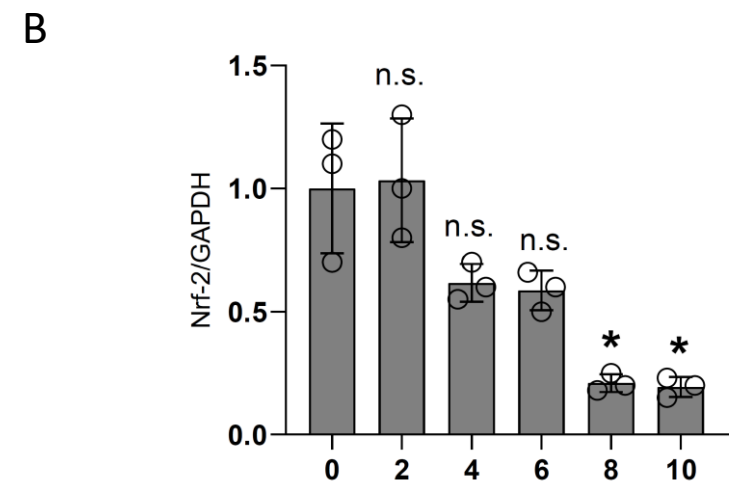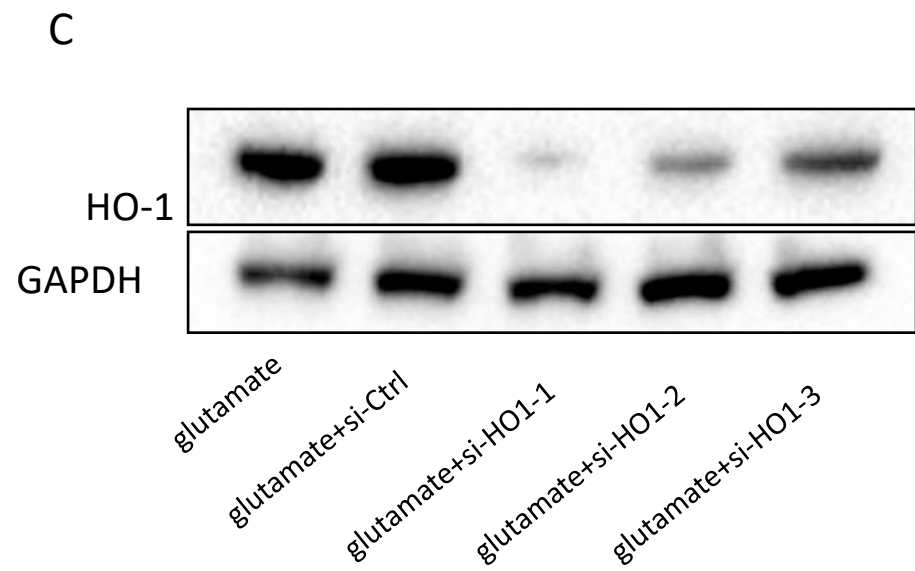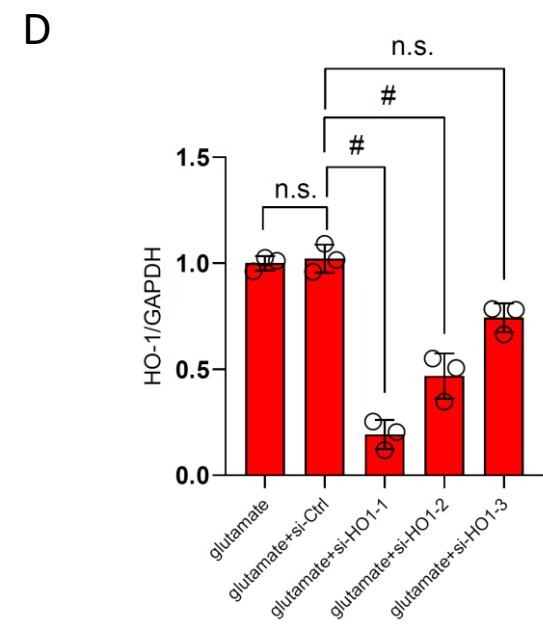

Supplement: Supplementary file 5 — Additional file 5: Fig. S5. A–D. Western blot analysis of Nrf-2 or HO-1 expression after transfected with ML385 or siHO-1, GAPDH were used as the loading control. And bar graphs show the results of analysis (by band density analysis) of these proteins (n = 3). Data are presented as the means ± SD; *P < 0.05 vs. control, #P < 0.05 vs. Glu + siCtrl group, and n.s.: no significant difference [file 12974_2022_2633_MOESM5_ESM.pdf]

A

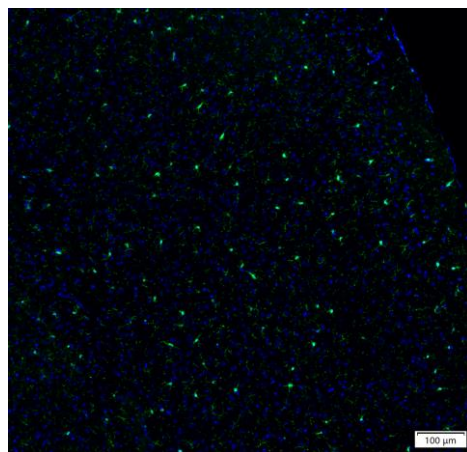

sham

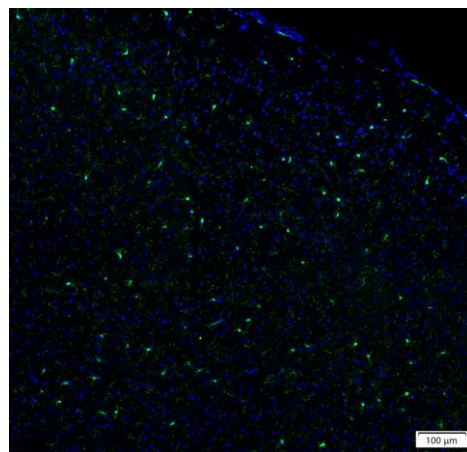

sham + Veh

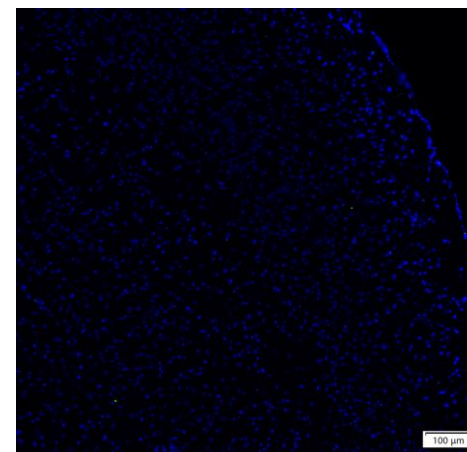

sham + PLX

B

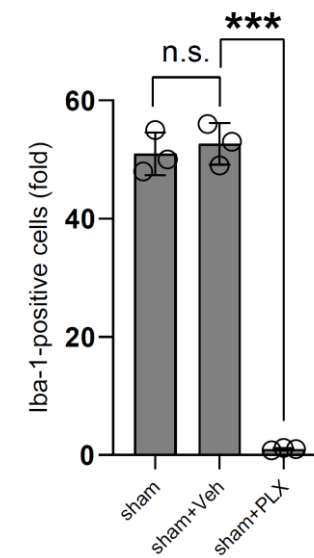

C

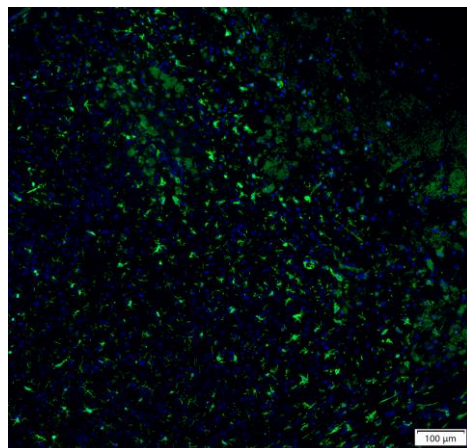

CCI

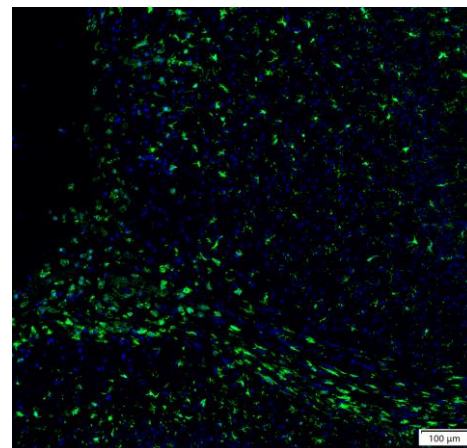

CCI + Veh

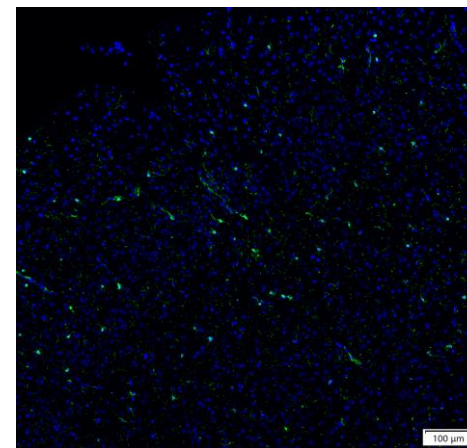

CCI + PLX

D

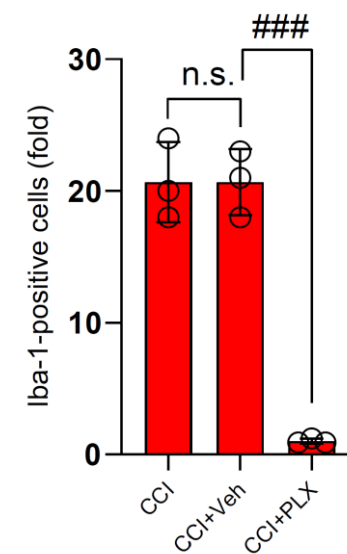

Supplement: Supplementary file 6 — Additional file 6: Fig. S6. PLX5622, which was specific CSF1R inhibitor, was able to achieve robust brain-wide microglia elimination. We fed PLX5622 to mice and tested its elimination efficiency. A, B. Immunofluorescence for Iba-1 in the hemisphere and statistical analysis of Iba-1. Scale bar is 100 μm (n = 3). Data are presented as the means ± SD; ***P < 0.001 vs. sham + Veh group, ###P < 0.001 vs. CCI + Veh group, and n.s.: no significant difference [file 12974_2022_2633_MOESM6_ESM.pdf]
